# Evaluation of Plant-Based Milks in Vegan Muffins: Functional, Structural, Rheological and Nutritional Characterization

**DOI:** 10.3390/foods14233989

**Published:** 2025-11-21

**Authors:** Kübra Topaloğlu Günan, Perihan Yolci Ömeroğlu

**Affiliations:** 1 Food Engineering Department, Faculty of Agriculture, Bursa Uludag University, Gorukle Campus, 16059 Bursa, Türkiye; kubratopaloglu@maltepe.edu.tr; 2 Department of Gastronomy and Culinary Arts, Faculty of Fine Arts, Maltepe University, 34857 Istanbul, Türkiye

**Keywords:** muffins, plant-based milk, milk substitute, vegan

## Abstract

As the demand for dairy-free bakery products increases, identifying plant-based milk alternatives that sustain product quality is essential. This study investigated the effects of eight milk types—soy, hazelnut, walnut, quinoa, flaxseed, coconut, oat, and almond—on the functional, nutritional, and sensory properties of muffins. A control prepared with cow’s milk served as reference. Rheological results showed that quinoa- and flaxseed-based batters exhibited stronger viscoelastic behavior, whereas oat and coconut milks reduced consistency. Physical parameters such as baking loss, volume index, and symmetry revealed no significant structural differences (*p* > 0.05), confirming that milk substitution did not affect baking performance. Color analysis indicated distinct chromatic variations, particularly in almond and coconut muffins with higher color difference (*ΔE*) and hue values. Phenolic and antioxidant assays demonstrated enhanced total phenolic content and Cupric ion reducing antioxidant capacity (CUPRAC) activity in quinoa and coconut variants. CUPRAC activity reached 0.89 micromoles Trolox equivalents per gram (µmol TE/g) in almond and 0.63 µmol TE/g in control muffins, whereas oat and hazelnut muffins exhibited the lowest activities, with 0.37 and 0.44 µmol TE/g, respectively. Amino acid profiling showed elevated glutamic acid and arginine in walnut, nearly doubling the control. Sensory scores (≥5) indicated high acceptability, confirming that selected plant-based milks can replace cow’s milk while enhancing functional and bioactive quality.

## 1. Introduction

Milk is a vital source of high-quality proteins, vitamins, minerals, and bioactive compounds essential for human nutrition and health [1]. However, its consumption has declined in recent years due to lactose intolerance; milk protein allergy; and concerns over saturated fat, cholesterol, and production-related residues [2]. Moreover, the growth of vegan and environmentally conscious lifestyles has intensified interest in non-dairy alternatives [2,3].

In this context, plant-based milk (PBM) alternatives have gained remarkable global attention as aqueous extracts of plant sources and are designed to mimic the sensory characteristics of conventional milk, including appearance, aroma, mouthfeel, and flavor [4]. PBM typically produced from vegetables, legumes, cereals, pseudo-cereals, and nuts through grinding, extraction, and homogenization processes, yielding suspensions with particle sizes of approximately 5–20 μm [5]. This structure allows them to resemble bovine milk in terms of texture and visual appearance. According to the classification proposed by Paul et al. plant-based milks can be grouped into five main categories: nut-based, seed-based, cereal-based, legume-based, and pseudo-cereal-based [6].

Beyond their structural resemblance, PBMs also provide valuable nutritional components. For instance, soy, peanut, hemp, and almond milk alternatives are rich sources of essential fatty acids, particularly linoleic and oleic acids, thereby offering significant health benefits [7,8]. Almond-based PBMs are additionally notable for their calcium and vitamin E content [7]. From a sensory perspective, product-specific differences have been reported: hemp, lentil, and soybean milks generally receive lower acceptance ratings, whereas oat, rice, and almond milks are evaluated more favorably [9]. Almond-based products, in particular, stand out in terms of mouthfeel and overall acceptance, largely due to their relatively higher fat content [10].

Muffins are widely appreciated by consumers for their versatility and adaptability, allowing the development of a broad range of flavors spanning from sweet to savory. They also serve as effective carriers for nutrients and bioactive compounds, while offering a relatively favorable nutritional profile compared with many other snack foods, which contributes to their continued popularity [11]. From a technological perspective, muffin batter represents a complex system composed of interacting ingredients such as flour, sugar, fat, eggs, and leavening agents, while additional components, including emulsifiers, preservatives, and milk powder, are often incorporated to improve texture, stability, and shelf life [11,12]. Milk fats act as tenderizing agents that reduce starch–gluten interactions, promote a finer crumb, and stabilize air cells during baking, while also enhancing flavor and aroma [13]. In line with the rising demand for plant-based products, alternative proteins such as lentil or soy isolates have been investigated as substitutes for milk and egg proteins in bakery formulations, yielding promising structural and sensory results [9,14].

A considerable number of studies have focused on the development of vegan cake formulations, in which various egg replacers have been tested and shown to produce favorable effects on the physicochemical properties of muffins [15,16,17]. Plant-based milks have also been widely investigated for their rheological and nutritional properties, particularly in the development of dairy alternatives such as ice cream, yogurt, and beverages [4,8,18,19]. However, research examining their effects in bakery systems remains limited, particularly in cakes, pancakes, and muffins [14,20]. Recent research has further highlighted the technological potential of plant-based milks in bakery applications. For instance, one study demonstrated that vegan muffins prepared with black chickpea flour, aquafaba, and almond milk achieved desirable textural and sensory properties [21]. Another study, a study reported that cakes made with soy or lupin milk exhibited distinct compositional and physical variations: soy milk–based cakes contained higher ash, fat, total phenolic content, antioxidant activity, and mineral levels, whereas increasing the legume milk ratio decreased volume index and increased hardness [22]. Despite the growing interest in plant-based nutrition, the functional implications of plant-based milks in bakery systems remain insufficiently understood. Most previous studies have focused on their nutritional composition or sensory appeal in beverage and dairy analog formulations, while comprehensive evaluations of their rheological, structural, and bioactive impacts in baked products are scarce. Addressing this gap is essential to determine how the compositional diversity of plant-based milks translates into differences in batter behavior, texture, and overall muffin quality. The present study therefore sought to address this gap by developing and evaluating vegan muffin formulations prepared with diverse plant-based milk alternatives. In our previous investigation on vegan cake formulations, several egg replacers were evaluated, and aquafaba was identified as a particularly promising ingredient, achieving an optimal balance between structural performance and consumer acceptability. Consistent with these findings, aquafaba was selected in the present study as the egg substitute owing to its demonstrated ability to enhance the textural and sensory properties of baked products [15,23]. The plant-based milk alternatives tested included soy milk (legume-based), oat milk (cereal-based), quinoa and flaxseed milks (pseudo-cereal- and seed-based, respectively), and nut-based varieties such as hazelnut, coconut, almond, and walnut milk. In this study, vegan muffins are defined as products made entirely from plant-based ingredients without any animal-derived components.

Specifically, the research aimed to comprehensively investigate the effects of these milk alternatives on muffin batter rheology, textural properties, volume, and moisture retention, as well as on nutritional characteristics including macronutrient composition, amino acid and fatty acid profiles, total phenolic content, antioxidant capacity, and in vitro bioaccessibility. Sensory evaluation and a 30-day shelf-life study were additionally conducted to assess consumer acceptance and product stability. Furthermore, chemometric modeling using principal component analysis (PCA) and hierarchical cluster analysis (HCA) was applied to provide multidimensional insights into the impact of each milk alternative. By integrating functional, nutritional, and sensory perspectives, this study supports the creation of bakery products that are sustainable, less processed, and nutritionally improved through the use of natural and innovative ingredients.

## 2. Materials and Methods

### 2.1. Materials

Eight different plant-based milk alternatives were evaluated in this study: soy, hazelnut, coconut, oat, almond, walnut, quinoa, and flaxseed milk. Among these, soy, hazelnut, coconut, oat, and almond milks (Alpro, Ghent, Belgium) were purchased as commercially available products from local markets in Istanbul, Türkiye. Their nutritional composition and ingredient lists were reported according to the information declared on product labels. In general, these products consisted of water, the respective plant ingredient (2–8%), stabilizers (e.g., gellan gum, locust bean gum), emulsifiers, salt, and fortifying agents such as calcium and vitamins (B2, B12, D, and E). In contrast, walnut, quinoa, and flaxseed milks were prepared in the laboratory following a standardized procedure, as these alternatives are not widely available commercially and were included to ensure compositional consistency and represent underexplored milk matrices.

Walnut (*Juglans regia* L., Tadım, Istanbul, Türkiye), quinoa (*Chenopodium quinoa* Willd., Yayla, Istanbul, Türkiye, origin: Peru) and flaxseed (*Plantago ovata* Forssk., Yayla, Ankara, Türkiye, origin: China) milks were prepared following a standardized procedure. Walnut milk was produced by soaking kernels in distilled water (1:4 *w*/*v*, 50 °C, 250 g walnut with 1000 mL water) for 12 h at room temperature (25 ± 2 °C) to soften them, followed by draining and homogenization. After soaking, the walnuts were blended with six times their weight of distilled water at 50 °C for 5 min using a high-speed blender (Zwilling Enfinigy, 1.4 L, Solingen, Germany). Quinoa seeds and flaxseeds, without prior soaking, were directly blended with eight times their weight of distilled water at 50 °C for 5 min using the same blender. The resulting slurries from the laboratory-prepared milks (walnut, quinoa, and flaxseed) were filtered through a 200-mesh sieve, heated to 90 °C for 10 min, cooled to 40 °C, then transferred into glass bottles and stored at 4 °C until further use [24,25,26,27].

Aquafaba was prepared from chickpeas following the standardized method previously described by Topaloğlu Günan and Yolci Ömeroğlu [15]. Briefly, chickpeas were soaked, pressure-cooked, and the resulting cooking liquid was collected and whipped to obtain a stable foam.

The muffin formulations were prepared using wheat flour (Sinangil, Istanbul, Türkiye), granulated sugar (Balküpü, Istanbul, Türkiye), sunflower oil (Yudum, Istanbul, Türkiye), baking powder, and vanillin (Dr. Oetker, Izmir, Türkiye). All ingredients were kept in their original packages under ambient conditions (22–24 °C, <50% relative humidity) until further use.

### 2.2. Chemicals and Reagents

All reagents and solvents were of analytical or chromatographic grade. Chemicals used for proximate composition and mineral analysis (e.g., petroleum ether, hydrochloric acid, nitric acid, hydrogen peroxide) were obtained from Merck (Darmstadt, Germany) and used according to AOAC and NMKL procedures. Enzymatic digestion for total dietary fiber measurement was performed using heat-stable α-amylase, protease, and amyloglucosidase provided in the Megazyme Total Dietary Fiber Kit (Bray, Ireland). Calibration standards were obtained by diluting a certified multi-element ICP stock solution (Standard VI, Merck, Darmstadt, Germany) with 1% nitric acid prepared using ultrapure water (18.2 MΩ·cm, Milli-Q, Millipore, USA). The solutions were stored at 4 °C in acid-cleaned polyethylene containers until analysis. High-purity argon gas (99.99%) served as the plasma source during ICP-MS measurements. For amino acid analysis, hydrochloric acid (Merck, Darmstadt, Germany), phenol (2 mM), and diethylenetriaminepentaacetic acid (DTDPA, 2%, Sigma-Aldrich, St. Louis, MO, USA) were used during hydrolysis to prevent oxidation and ensure recovery of sulfur-containing amino acids. Amino acid standards (Dr. Ehrenstorfer, Augsburg, Germany) were used for calibration, and ultrapure water (Milli-Q, Millipore, Molsheim, France) was used throughout the analyses. For in vitro digestion and bioaccessibility assays, α-amylase (1500 U/mL), pepsin (25,000 U/mL), pancreatin (800 U/mL), and bile extract (porcine, Sigma-Aldrich, USA) were used to simulate oral, gastric, and intestinal phases following the standardized in vitro digestion model. Calcium chloride (Tekkim, Türkiye) and sodium hydroxide (1 M, Merck, Germany) were employed for pH adjustment. For spectrophotometric analyses, Folin–Ciocalteu reagent, sodium carbonate, and gallic acid (Merck, Germany) were used to quantify total phenolic content (TPC), while total flavonoid content (TFC) was determined using aluminum chloride, sodium nitrite (Merck, Germany), and sodium hydroxide (Sigma-Aldrich, USA), with rutin (Acros Organics, Waltham, MA, USA) as the reference standard. Unless otherwise indicated, all other solvents and reagents, such as ethanol and acetonitrile, were obtained from Merck (Germany) and Sigma-Aldrich (USA). For fatty acid composition analysis, a 37-component fatty acid methyl ester (FAME) standard mixture (Supelco, Sigma-Aldrich, Darmstadt, Germany) was used as the reference. The stock solution was stored at −20 °C and diluted in chloroform before use. Methylation and extraction were performed using boron trifluoride–methanol solution (14%, Sigma-Aldrich, USA) and n-hexane (Merck, Germany), respectively, and nonadecanoic acid methyl ester (C19:0, Sigma-Aldrich, USA) served as the internal standard.

### 2.3. Methods

#### 2.3.1. Muffin Preparation

Muffin production was carried out following the method described by Topaloğlu Günan and Yolci Ömeroğlu [15] with modifications to incorporate aquafaba and different plant-based milk alternatives. The formulation of the muffins is presented in Table 1. For all experimental samples, aquafaba was used as the sole egg substitute at an equivalent weight of 90 g per formulation, based on previous studies reporting comparable foaming and emulsifying properties [28]. Each muffin formulation was prepared by substituting cow’s milk in the control batter with an equivalent amount (32 g) of plant-based milk (soy, hazelnut, walnut, quinoa, flaxseed, coconut, oat, or almond). The foaming mixture was initiated by whipping aquafaba together with granulated sugar in a stand mixer fitted with a whisk attachment, operated at 220 rpm for 4 min. Subsequently, sunflower oil and the selected plant-based milk were incorporated and blended at 135 rpm for 1 min. The dry constituents were then added, and mixing continued at 95 rpm for a further minute until a uniform batter was achieved.

Approximately 40 g of batter was portioned into each cavity of a 12-cavity muffin pan (Zenker, Aichach, Germany; 7.0 × 5.6 cm). Baking was carried out in a preheated convection oven (Inoksan, Bursa, Türkiye) at 170 ± 2 °C for 16 min under fan-assisted conditions. Following baking, samples were left to cool inside the molds at ambient temperature (25 ± 2 °C) for about 30 min prior to demolding. Three independent batches were produced for each milk variant to ensure experimental reproducibility.

#### 2.3.2. Physicochemical and Technological Attributes of Muffins

After baking, muffins were cooled in their trays at ambient temperature (25 ± 2 °C) for 30 min and then carefully removed. To ensure consistency across physical and structural measurements, all samples were left to equilibrate at room temperature for 24 h before testing. The physical indices evaluated included volume index (VI), symmetry index (SI), and uniformity index (UI), together with baking loss, texture, color, and microstructural characteristics.

The VI, SI, and UI values were determined with a digital caliper (accuracy ±0.01 mm) by measuring three predefined positions on the muffin surface, according to the AACC 10–91 method [29]. The VI, representing average product height, was calculated as shown in Equation (1):

(1) $$VI=\frac{A+B+C}{3}\;(mm)$$

where A corresponds to the center height, while B and C represent the left and right edges. The SI was obtained from the absolute difference between the edge measurements, as given in Equation (2):

(2) SI=│B−C│ (mm)

An SI ≤ 5 mm indicates acceptable symmetry, while larger deviations suggest uneven baking. UI, expressing the uniformity of the surface rise, was calculated as in Equation (3):

(3) $$UI=\frac{│A-B│+│A-C│}{2}\;(mm)$$

Values ≤ 5 mm were considered satisfactory, whereas higher readings implied non-uniform doming or collapse. Baking loss was calculated by comparing the batter weight before baking (wd) with the cooled product weight (wc), as given in Equation (4) [30]:

(4) $$Baking\;Loss=\frac{wd-wc}{wd}\times 100\;(\%)$$

Texture profile analysis was conducted with a Stable Micro Systems TAXTPlus analyzer. Muffins stored for 24 h were cut into 2.5 cm cubes [31]. A double-compression test was performed using a 36 mm cylindrical probe with 40% deformation at 1.0 mm/s, a 5 kg load cell, and a 5 g trigger force. Five parameters were assessed: hardness, springiness, cohesiveness, chewiness, and resilience [32].

The rheological behavior of the muffin batters was analyzed under steady shear conditions (0.01–100 s^−1^) using a controlled shear rate protocol. Experimental shear stress–shear rate data were described by the Power Law model (Equation (5)):

(5) $$\tau =K{\dot{\gamma }}^{n}$$

where *τ* represents the shear stress (Pa), *K* is the consistency coefficient (Pa·s^n^), *n* denotes the flow behavior index (dimensionless), and $\dot{\gamma }$ is the applied shear rate (s^−1^).

Oscillatory rheological measurements were conducted on an Anton Paar MCR302 rheometer (Austria) equipped with a 50 mm parallel plate system and a 0.5 mm measurement gap at 25 °C. Amplitude sweeps (0.01–100% strain, 1 Hz) identified the linear viscoelastic region, which for all batters ranged from 0.1% to 10% strain. Frequency sweeps (0.1–10 Hz) were then performed at 0.1% strain to determine *G*′ (storage modulus) and *G*″ (loss modulus). Data were modeled according to the power law Equations (6) and (7):

(6) $$G^{\prime }=K^{\prime }{\left(\omega \right)}^{n^{\prime }}$$

(7) $$G^{\prime \prime }=K^{\prime \prime }{\left(\omega \right)}^{n^{\prime \prime }}$$

where *K*′ and *K*″ are consistency indices (Pa.s^n^), *n*′ and *n*″ are frequency exponents, and ω is angular frequency (rad s^−1^). The viscoelastic balance was further described by the loss tangent (tan *δ* = *G*″/*G*′) at 1.36 rad s^−1^.

The color characteristics of the muffin crust and crumb were evaluated using a chroma meter (CR-400, Minolta, Osaka, Japan) operating in the CIELab* color space. In this system, L* represents brightness (ranging from 0 for black to 100 for white), while a* and b* correspond to the red–green and yellow–blue coordinates, respectively. The internal crumb color was measured on vertical cross-sections. Chroma (C*) and hue angle (*h*°) were calculated using Equations (9) and (10) [33]. The total color difference (*ΔE*) between samples and the control was calculated determined as Equation (8).

(8) $$\Delta E=\sqrt{{(L}^{*}-L_{0}^{*}{)}^{2}+{{(a}^{*}-a_{0}^{*})}^{2}+{(b}^{*}-b_{0}^{*}{)}^{2}}$$

(9) $$C^{*}=\sqrt{{(a}^{*2}+L^{*2})}$$

(10) $$h^{o}=arctan\left(\frac{b}{a}\right)$$

where *L*_0_*, *a*_0_*, and *b*_0_* are the reference values. The hue angle was interpreted as: 0–90° (a* > 0, b* > 0), 90–180° (a* < 0, b* > 0), 180–270° (a* < 0, b* < 0), 270–360° (a* > 0, b* < 0).

The microstructure of muffin samples was analyzed with a field-emission scanning electron microscope (Apreo S; Thermo Scientific, Waltham, MA, USA). Freeze-dried and fractured samples were gold-coated and imaged under high-vacuum conditions at magnifications of 100× and 250×.

#### 2.3.3. Chemical Composition

Moisture content (%) was determined according to the AOAC procedure [34] and ash content (%) was analyzed following the method described in [35], by incinerating the samples at 550 °C in a muffle furnace.

Protein content (%) was determined using the Dumas combustion technique with a nitrogen analyzer, following the AOAC procedure [31]. The nitrogen content obtained was converted to protein by applying a conversion factor of 6.25. Total dietary fiber (%) was quantified following the enzymatic–gravimetric procedure described by AOAC [32].

Total fat (%) was determined according to NMKL method No. 160 [36]. Carbohydrate content (%) was calculated by difference using Equation (11) [37]:

(11) $$Carbohydrate\left(\%\right)=100-(Moisture+Ash+Protein+Fat+Dietary\;Fiber)$$

The energy content (kcal/100 g) was calculated according to the Atwater system recommended by the FAO [37]. Conversion factors for macronutrients were applied as shown in Equation (12). All proximate composition results are presented on a wet weight basis.

(12) Energy(kcal/100g)=(Protein×4)+(Fat×9)+(Carbohydrate×4)+(Dietary Fiber×2)

Mineral elements (Ca, K, Mg, Na, and P) were determined by inductively coupled plasma–mass spectrometry (ICP-MS) following the NMKL No. 186 method [38]. All measurements were conducted in triplicate and expressed on a dry weight basis.

#### 2.3.4. Fatty Acid Composition

Fatty acid methyl esters (FAMEs) were prepared using the trans-methylation procedure under alkaline conditions in accordance with ISO guidelines (ISO, 2014a–c [39,40,41]), with minor modifications. Briefly, 0.1 g of extracted fat from each sample was transferred into a 2 mL screw-capped test tube and dissolved in 2 mL of heptane by vigorous shaking. For methylation, 0.2 mL of 2 N potassium hydroxide in methanol was added, and the mixture was centrifuged at 4500–5000× *g* rpm (Sigma 2–16 P, Osterode, Germany). The clear upper phase containing FAMEs was collected and transferred into GC vials. If analysis was not performed immediately, samples were kept at 4 °C for no longer than 12 h.

The FAME composition was determined using a gas chromatograph (Agilent 7890 A, Santa Clara, CA, USA) equipped with a flame ionization detector (FID) and an HP-88 capillary column (100 m × 0.25 mm i.d., 0.20 μm film thickness; Agilent, Santa Clara, CA, USA). Analyses were performed with a split injection ratio of 50:1. Helium was used as the carrier gas at 29.06 mL/min, while hydrogen (30 mL/min) and dry air (400 mL/min) served as flame gases. Injector and detector temperatures were set at 250 °C and 255 °C, respectively. The oven temperature program was as follows: initial temperature of 130 °C, ramped to 170 °C at 4 °C/min, then increased to 215 °C at 1.7 °C/min and held for 20 min, followed by a final increase to 240 °C at 10 °C/min and held for 1 min. The injection volume was 0.2 μL, and analyses were carried out under constant pressure at 242 kPa.

Individual fatty acids were identified by comparing their retention times with those of certified FAME reference standards. Quantification was based on peak area normalization, expressed as the relative percentage of each FAME to the total peak area. Fatty acids were further grouped into saturated fatty acids (SFA, no double bonds), monounsaturated fatty acids (MUFA, one double bond), polyunsaturated fatty acids (PUFA, two or more double bonds), and total unsaturated fatty acids (TUFA), following the classification of Omeroglu and Ozdal [42]. Total amounts of SFA, MUFA, PUFA, and TUFA were calculated as the sum of the respective fatty acids, and expressed both as percentage of total fatty acids and as g/100 g sample, considering the overall fat content.

#### 2.3.5. Phenolic Extraction, In Vitro Digestion Model, Total Antioxidant Capacity (TAC) and Total Phenolic Content (TFC) Determination

Phenolic compounds were analyzed in both the muffin samples and the milk types used in their preparation. Extraction, in vitro digestion, and antioxidant assays (TPC, DPPH, and CUPRAC) were conducted according to the procedures described by Topaloğlu Günan and Yolci Ömeroğlu [15], with minor modifications related to solvent volume and sample size. Total phenolic content (TPC) was determined using the Folin–Ciocalteu method, while total antioxidant capacity (TAC) was assessed by the DPPH and CUPRAC assays. Results were expressed as mg gallic acid equivalents (GAE) per 100 g for TPC and mg Trolox equivalents (TE) per kg dry matter for TAC. The bioaccessibility of phenolic compounds was calculated as the percentage ratio between post-digestion and undigested values.

#### 2.3.6. Amino Acid (AA) Composition

The amino acid composition of the muffin samples was determined using a high-performance liquid chromatography (HPLC) system (Agilent Technologies, Santa Clara, CA, USA) equipped with a diode-array detector. The analytical procedure followed the method described by Topaloğlu Günan and Yolci Ömeroğlu [15], with slight modifications in sample weight and solvent ratios. Briefly, both free and total amino acids were quantified after appropriate extraction and acid hydrolysis steps. Pre-column derivatization was performed using o-phthalaldehyde (OPA) and fluorenylmethyloxycarbonyl chloride (FMOC) reagents, and chromatographic separation was achieved on a ZORBAX Eclipse AAA column under gradient elution. Quantification was based on calibration with certified amino acid standards, and results were expressed as grams of amino acid per 100 g of sample. Both essential and non-essential amino acids were included in the profile.

#### 2.3.7. Shelf Life Analysis

Shelf-life evaluation was performed over a 30-day storage period under ambient conditions (25 ± 2 °C, 60 ± 5% RH). Physicochemical parameters, including moisture, fat, texture, and color, were monitored on days 0, 15, and 30 following standard analytical procedures [43].

Microbiological quality was also examined at the same intervals to assess product safety and stability. Samples were analyzed for total aerobic mesophilic bacteria, molds and yeasts, *Escherichia coli*, *Salmonella* spp., and *Staphylococcus aureus* using conventional plating methods and selective media, as previously described by Topaloğlu Günan and Yolci Ömeroğlu [15]. All analyses were conducted under aseptic conditions, and results were interpreted according to internationally accepted microbiological standards for bakery products.

#### 2.3.8. Sensory Evaluation of Muffins

A descriptive sensory evaluation was performed with a trained panel consisting of 20 participants (12 females and 8 males) aged 22–49 years. All assessors were either undergraduate or academic members of the Department of Gastronomy and Culinary Arts at Maltepe University and were familiar with bakery products. Prior to testing, the panel received instruction consistent with international sensory evaluation guidelines [44,45,46]. Each muffin sample was coded with randomly generated three-digit numbers and served on neutral plastic plates to prevent bias. Evaluations covered seven sensory parameters: crust color, crumb color, aroma, flavor, appearance, softness, and overall acceptability. Panelists rated each attribute using a nine-point hedonic scale, where 1 corresponded to “extremely poor” and 9 to “excellent.” Samples achieving average scores of 5 or higher were considered acceptable.

All assessments were carried out in individual booths maintained at 22 ± 1 °C under uniform, neutral lighting. To avoid flavor carryover between evaluations, panelists cleansed their palates with room-temperature water and unsalted breadcrumbs. Ethical approval for the study was obtained from the Maltepe University Ethics Committee (Approval No. 2024/22-09, dated 28 November 2024).

#### 2.3.9. Statistical Analysis

Unless otherwise specified, all analyses were conducted in triplicate (*n* = 3). The sensory evaluation was performed by a panel of 20 trained assessors, each evaluating the samples once under controlled laboratory conditions. The overall formulation and experimental design are outlined in Table 1. Results are expressed as mean ± standard deviation.

Statistical analyses were performed using SPSS software (version 21.0; SPSS Inc., Chicago, IL, USA). The effect of milk type on each parameter was assessed through one-way analysis of variance (ANOVA, version 21.0), and significant differences among means were identified using Tukey’s post hoc test (*p* < 0.05). Relationships among physicochemical, structural, nutritional, and bioactive parameters were examined using Pearson’s correlation coefficients. To explore patterns and similarities among the samples, multivariate techniques were also applied. Principal component analysis (PCA) was used to visualize clustering tendencies among formulations, whereas hierarchical cluster analysis (HCA) was conducted based on Ward’s linkage and Euclidean distance criteria. Both analyses were carried out in SPSS, and the resulting graphical outputs were further refined in OriginPro 2025 to improve visual clarity.

## 3. Results and Discussion

### 3.1. Rheological Characterization of Muffin Batters

**Figure 1:**
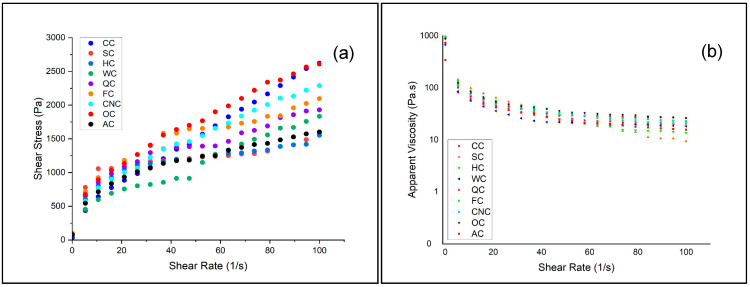
Flow behavior of muffin samples: (**a**) Shear stress vs. shear rate, (**b**) Apparent viscosity vs. shear rate. CC: Control muffin, SC: Soy milk muffin, HC: Hazelnut milk muffin, WC: Walnut milk muffin, QC: Quinoa milk muffin, FC: Flaxseed milk muffin, CNC: Coconut milk muffin, OC: Oat milk muffin, AC: Almond milk muffin.

The rheological characteristics of muffin batters were analyzed using a controlled-stress rheometer to evaluate how different milk alternatives influenced flow behavior and viscoelastic properties (Figure 1).

The viscosity of muffin batter is a critical determinant of product quality. Inappropriate viscosity may negatively influence aeration and texture during baking. Excessively high viscosity restricts gas bubble expansion, leading to dense and compact structures, while overly low viscosity causes structural collapse and insufficient crumb development. Therefore, a well-balanced viscosity is essential to ensure proper aeration, optimal volume, and stable texture in muffins [47]. The flow behavior of aquafaba-based muffin batters prepared with different milk types was investigated using steady-shear testing, and the results are presented in Figure 1a,b.

All samples exhibited non-Newtonian, shear-thinning behavior, characterized by a decrease in apparent viscosity with increasing shear rate. This pseudoplastic flow is typical for cake and muffin batters, where weak intermolecular bonds are disrupted under shear forces. However, clear differences were observed depending on the type of milk used. Batters prepared with coconut milk (CNC), oat milk (OC), and flaxseed milk (FC) demonstrated higher shear stress and viscosity values, indicating stronger internal cohesion and higher resistance to deformation. This effect may be attributed to the elevated lipid and soluble fiber fractions in these plant-based milks, which increase water-binding capacity and contribute to a more viscous batter matrix [48,49,50]. In contrast, almond milk (AC) and hazelnut milk (HC) resulted in lower shear stress and viscosity across the tested range, reflecting weaker structural networks and more fluid-like consistency [51,52]. Quinoa milk (QC) and walnut milk (WC) showed intermediate values, suggesting that their protein and mineral components provided partial reinforcement of batter structure (Table 4). The control batter prepared with cow’s milk (CC) exhibited intermediate behavior, positioned between the high-viscosity and low-viscosity formulations.

These observations are consistent with previous reports showing that batters enriched with hydrocolloids or fiber-rich plant ingredients exhibit higher viscosity and improved stability, while formulations with lower solids or weaker emulsifying capacity tend to produce more fluid systems [53,54,55]. The shear-thinning behavior observed across all formulations is technologically advantageous, as it facilitates air incorporation during mixing and supports gas retention during baking. However, the magnitude of viscosity strongly depends on milk composition, highlighting the role of plant-based milk type in determining structural reinforcement and flow behavior of aquafaba-based muffin batters. The results demonstrate that milk alternatives with higher fiber or lipid fractions (coconut, oat, flaxseed) enhance batter consistency, while nut-based milks (almond, hazelnut) yield weaker networks. This indicates that the choice of milk significantly influences the rheological performance of aquafaba-based muffin formulations, with direct implications for aeration, expansion, and final product texture.

**Figure 2:**
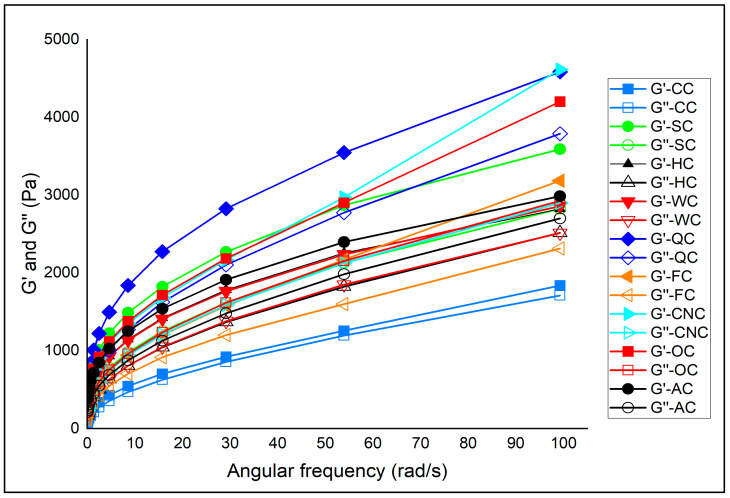
The rheological parameter of batter sample, elastic modulus (*G*′) and viscous modulus (*G*″). CC: Control muffin, SC: Soy milk muffin, HC: Hazelnut milk muffin, WC: Walnut milk muffin, QC: Quinoa milk muffin, FC: Flaxseed milk muffin, CNC: Coconut milk muffin, OC: Oat milk muffin, AC: Almond milk muffin.

The viscoelastic behavior of muffin batters prepared with aquafaba and different milk alternatives was investigated by frequency sweep tests, and the results are shown in Figure 2 and Table 2. Frequency sweep measurements are crucial for understanding batter performance, as they provide information on the balance between elastic (*G*′) and viscous (*G*″) moduli. A predominance of *G*′ over *G*″ indicates solid-like behavior and a more stable network, which is desirable for gas retention and structure setting during baking. Conversely, higher *G*″ or tan *δ* values point to weaker elastic networks and greater viscous character, which may compromise batter stability and product texture [56].

Across all formulations, both *G*′ and *G*″ increased with frequency, confirming the weak gel-like and shear-dependent nature of muffin batters. Importantly, *G*′ values were consistently higher than *G*″ in all samples, indicating that elastic contributions dominated over viscous losses, which is characteristic of stable bakery batters [57].

Among the samples, coconut milk (CNC) exhibited the highest *K*′ value (1838.30 Pa.s^n^) together with a relatively high *K*″, indicating that CNC batters possessed stronger elastic resistance and enhanced structural reinforcement compared to other formulations [58]. Quinoa (QC) and oat milk (OC) batters also showed high *K*′ values (1671.60 and 1706.20 Pa.s^n^, respectively), reflecting solid-like networks and good stability. By contrast, almond (AC) and hazelnut (HC) batters had lower *K*′ values (1235.10 and 1048.20 Pa.s^n^, respectively). The control sample with cow’s milk (CC) displayed the lowest *K*′ (598.32 Pa.s^n^), highlighting the reinforcing role of certain plant-based milks in aquafaba systems [59].

The loss factor (tan *δ*) provided further insights. Lower tan *δ* values (closer to elastic dominance) were observed in SC (0.609), OC (0.631), and CNC (0.636), indicating stronger elastic contributions and better stability. Conversely, higher tan *δ* values in FC (0.692) and CC (0.778) indicated a relatively greater viscous character, which may lead to reduced gas retention and less stable crumb structure [60]. Almond and hazelnut batters showed intermediate tan *δ* values (0.624 and 0.643, respectively), suggesting moderate viscoelastic balance but weaker networks compared to CNC or OC.

These findings align with previous studies indicating that the presence of fiber- or hydrocolloid-rich constituents in plant matrices tends to increase *G*′ and decrease tan *δ*, thus improving batter stability and baking performance. The observed differences among formulations may therefore be partially attributed to the compositional variation in the plant-based milks used (e.g., solids, protein, and fiber contents) [55,60,61].

Frequency sweep analysis demonstrated that milk type significantly influenced the viscoelastic properties of aquafaba-based muffin batters. Coconut, oat, and quinoa milks promoted stronger elastic networks and lower tan *δ* values, enhancing batter stability and potentially improving gas retention during baking. Almond and hazelnut milks, however, contributed to weaker structures, indicating reduced elastic reinforcement. These rheological trends are expected to directly influence the textural attributes and volume development of the final baked products.

### 3.2. Physical Properties of Muffin Samples

**Figure 3:**
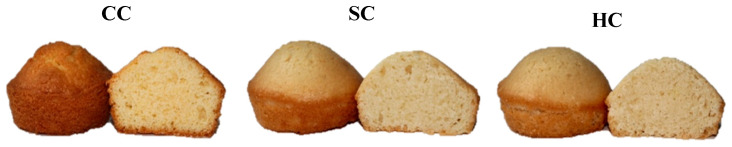

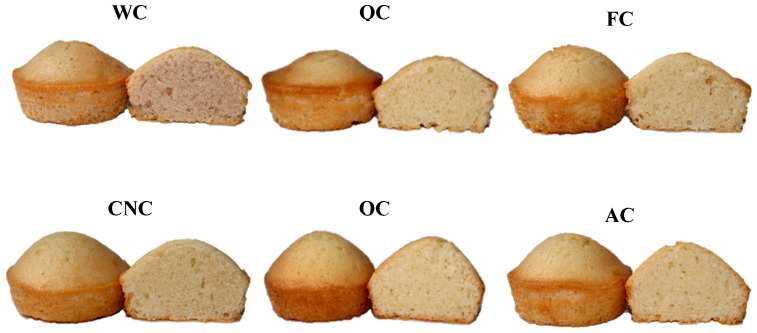
CC: Control muffin, SC: Soy milk muffin, HC: Hazelnut milk muffin, WC: Walnut milk muffin, QC: Quinoa milk muffin, FC: Flaxseed milk muffin, CNC: Coconut milk muffin, OC: Oat milk muffin, AC: Almond milk muffin.

The physical attributes and external appearance of the muffin samples are summarized in Table 3 and illustrated in Figure 3. Following baking, the percentage of baking loss varied between 5.63% in the AC sample and 6.91% in the OC sample. Although these variations were not statistically significant (*p* > 0.05), the slightly lower values observed in AC and CNC muffins suggest improved moisture retention during baking. In contrast, the higher loss recorded for the OC muffins may be associated with the presence of soluble fiber and β-glucans, which can modify water distribution and evaporation dynamics in the batter matrix.

Table 3 and Figure 3 summarize the muffins’ physical characteristics and visual appearance, respectively. After baking, baking loss ranged from 5.63% in the AC muffin to 6.91% in the OC muffin. Although the differences were not statistically significant (*p* > 0.05), slightly lower losses in AC and CNC muffins may indicate better water retention capacity, whereas OC exhibited higher loss, which may be related to its fiber and β-glucan content influencing water distribution during baking [62].

VI, a key indicator of muffin structural quality, was highest in the control sample (119.00 mm), consistent with the structural contributions of egg proteins. Plant-based milk alternatives are often reported to exhibit weaker gelation, water holding, and viscoelastic properties compared with cow’s milk, which could explain their tendency toward lower VI values [63]. Among plant-based variants, OC (102.33 mm), AC (100.67 mm), and HC (101.33 mm) exhibited satisfactory leavening performance, whereas WC had the lowest VI (91.00 mm). Although no formal threshold has been established for VI values in bakery products, measurements above 100 mm are generally regarded as indicative of favorable structural attributes. Similar findings have been reported in other plant-based systems, where aquafaba–milk combinations achieved VI values above 100 mm [15,21], while lentil protein–based muffins were able to maintain comparable volume indices despite complete substitution of egg and milk proteins [64]. These results highlight that, although plant-based proteins and flours often reduce structural expansion, optimized formulations can still yield VI values within the desirable range for good muffin quality. Statistical analysis confirmed that the VI values differed significantly among samples (*p* < 0.05), supporting the visual distinctions in muffin height (Figure 3).

SI, was determined from the height difference between the two lateral edges of the muffins, expressed as an absolute value. SI values ranged between 7.00 and 10.33 mm. The absence of negative readings indicates that none of the samples underwent structural collapse during baking [65]. According to Dadalı and Elmacı [66], cakes generally exhibit positive SI values, reflecting a higher central region compared to the edges as a consequence of gas expansion and structural stabilization during baking. Nevertheless, when SI values become excessively high, they may indicate surface irregularities, often arising from uneven batter distribution or thermal gradients within the oven. In the present study, the SC sample displayed the lowest SI value (7.33 mm), corresponding to the most uniform surface profile. By contrast, the AC and FC samples showed elevated SI values (≥10 mm), reflecting greater surface unevenness and irregularity. These variations were not statistically significant (*p* > 0.05), indicating that the type of plant-based milk substitute did not exert a measurable effect on surface symmetry.

UI, defined as the mean absolute difference between central and peripheral heights, serves as an indicator of lateral symmetry and surface regularity in muffins. Values approaching zero are desirable, as they reflect an even rise and a well-balanced surface profile. In this study, UI values ranged from −1.00 to 1.00 mm, with SC and FC samples exhibiting values of 0.00 mm. These findings are consistent with previous reports confirming that very low UI values correspond to uniform muffin morphology [66]. Statistical evaluation showed no significant variation among the formulations (*p* > 0.05), indicating that milk substitution had no discernible influence on muffin surface symmetry.

Muffin batters with higher storage modulus (*G*′) values tended to form firmer and more elastic networks, which in turn limited air incorporation and reduced the final muffin expansion. For instance, the coconut milk sample (CNC), which exhibited the highest *K*′ value (1838.30 Pa.s^n^), also showed relatively high hardness and a moderate VI, supporting the idea that excessive batter rigidity restricts bubble growth during baking. In contrast, almond (AC) and hazelnut (HC) batters, with comparatively low *K*′ values (1235.10 and 1048.20 Pa.s^n^, respectively), displayed weaker structures that permitted greater fluidity but limited the development of a stable muffin height. The control sample (CC), characterized by the lowest *K*′ (598.32 Pa.s^n^) and the highest tan *δ* (0.778), showed reduced elastic dominance. This indicates that cow’s milk proteins, when combined with aquafaba, were not able to form a viscoelastic network as strong as those observed in certain plant-based milk formulations. Previous studies have shown that aquafaba alone can establish elastic gel-like structures, while the presence of fiber- or hydrocolloid-rich plant ingredients (such as β-glucans or seed mucilages) further enhances *G*′ and reduces tan *δ*, thereby improving batter stability and baking performance [49,62,63]. These findings support the present observation that coconut, oat, and quinoa milks reinforced aquafaba-based batters more effectively than cow’s milk, highlighting the importance of milk composition in determining rheological behavior and final muffin quality.

The influence of different milk alternatives on muffin texture was investigated, and the corresponding results are summarized in Table 3. The data indicate that the type of milk used had a measurable effect on the textural attributes of the final product. The highest hardness values were recorded in SC (1616.01 gf) and HC (1584.69 gf), whereas the lowest were observed in WC (976.59 gf) and FC (1015.00 gf). Most plant-based milks increased hardness compared to CC, likely due to differences in protein composition and water distribution within the crumb [22]. Springiness measurements revealed that CC (0.91) exhibited the highest elasticity, while FC (0.84) and WC (0.86) showed reduced values. consistent with previous reports showing that egg or milk proteins provide superior elasticity compared to plant-based substitutes [56,60]. The highest cohesiveness was also found in CC (0.71), whereas SC, HC, FC, CNC, and AC samples exhibited lower cohesiveness (0.58–0.60). These results reveal that plant-based milk alternatives were less effective in maintaining internal structural binding. Regarding chewiness, CC (828.54), SC (823.56) and HC (832.87) showed the highest values, whereas FC (513.33) and WC (534.95) had the lowest. Resilience was highest in CC (0.33) and lowest in SC (0.25), HC (0.26) and AC (0.26), indicating that cow’s milk contributed to superior structural recovery compared to plant-based milks. In conclusion, the results indicate that milk alternatives substantially altered the textural characteristics of muffins, underlining the importance of optimizing formulations to achieve the desired texture profile.

Color is a critical indicator of muffin quality, largely governed by Maillard browning and caramelization during baking. The crust color arises mainly from reactions between reducing sugars and amino compounds, while the crumb color depends on the specific composition and pigmentation of the ingredients [14,28]. In this study, color parameters were measured using the CIELAB system for both crust and crumb portions, and the corresponding results are summarized in Table 3.

Crust brightness (L*) ranged from 58.98 (CC) to 69.12 (AC). The highest crust lightness was observed in AC, followed by CNC (67.46) and WC (65.12). In contrast, CC (58.98) showed the lowest value, which can be attributed to the pigments naturally present in egg yolk [67]. Similar increases in brightness have been reported in cakes formulated with plant-based proteins or fibers that enhance water binding and surface reflectance [21,68]. Crust a* values, representing redness, were highest in CC (12.48), whereas all plant-based variants showed significantly lower a* values. This reduction indicates that the absence of egg-derived pigments limited reddish hues [68]. Crust b* values followed a similar trend, with CC exhibiting the highest yellowness (30.99), while WC (24.57) and QC (24.49) displayed markedly lower values. Since internal muffin temperature does not exceed 100 °C, Maillard reactions are restricted, and observed color differences can be primarily attributed to raw ingredients [65]. The yellowish tone of the CC sample (Figure 3) can be attributed to the carotenoids present in egg yolk [69].

Crumb lightness (L*) was highest in CC (75.03) and lowest in WC (64.69), producing a noticeably darker crumb. Across all samples, the crumb appeared more reddish or yellowish than the crust. Notably, WC was the only sample with a positive crumb a* (1.22), indicating a distinct reddish tone, whereas CC and the other plant-based muffins displayed negative a* values. Crumb b* values were highest in CC (24.33) and lowest in WC (12.11). These differences may be attributed to arise from compositional factors in walnut milk, such as phenolic compounds and fiber, which can participate in Maillard or oxidation reactions and thereby contribute to a darker internal color [70].

Beyond the basic L*, a*, and b* parameters, Chroma (C*), hue angle (*h*°), and total color difference (*ΔE*) were also computed to provide a more comprehensive assessment of visual appearance. The control muffins (CC) exhibited the most intense coloration, with C* values of 33.49 for the crust and 24.43 for the crumb, whereas the walnut-based samples (WC) showed the lowest saturation (25.03 and 12.17, respectively). Hue angle evaluation indicated a gradual transition in crust color from orange-yellow in CC (67.88°) to brighter yellow tones in the flaxseed (FC, 81.01°) and almond (AC, 83.19°) muffins. In contrast, the crumb of CC showed a hue of 275.35°, corresponding to the blue-green region of the CIELAB color space—a result of negative a* combined with dominant positive b* coordinates. The largest total color deviations (*ΔE*) for the crust were observed in AC (18.20) and CNC (15.85), whereas WC demonstrated the greatest difference in crumb color (16.72). These outcomes clearly demonstrate that incorporating plant-based milks into muffin formulations leads to visually perceptible color modifications relative to the control sample [22].

### 3.3. Morphology

**Figure 4:**
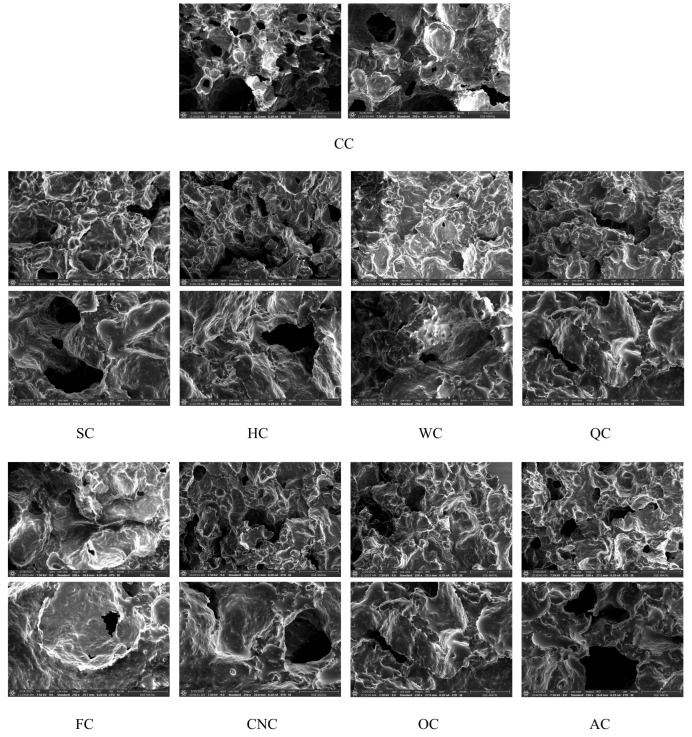
The microstructural features of the muffins were visualized by scanning electron microscopy (SEM) at 100× and 250× magnifications. CC: Control muffin, SC: Soy milk muffin, HC: Hazelnut milk muffin, WC: Walnut milk muffin, QC: Quinoa milk muffin, FC: Flaxseed milk muffin, CNC: Coconut milk muffin, OC: Oat milk muffin, AC: Almond milk muffin.

In this study, the effects of different milk alternatives on the microstructure of muffins were examined using scanning electron microscopy (SEM) (Figure 4).

The CC sample exhibited a continuous and homogeneous protein–starch matrix with uniformly distributed pores. By contrast, SC and HC samples showed denser and more compact structures with reduced pore size. Similar compact microstructures have been reported for plant-protein- or nut-derived ingredients in cakes, where increased solids and protein–lipid interactions limit gas-cell expansion [68,71]. WC and QC samples exhibited relatively larger and more irregular pores compared with CC. Such pore morphology is generally associated with weaker network stabilization and heterogeneous gas-cell distribution, as reported in cakes formulated with walnut flour and quinoa flour [72,73]. FC exhibited an irregular and collapsed pore structure, likely linked to its mucilage content increasing viscosity and restricting uniform air cell distribution, consistent with reports of structural collapse in aquafaba cakes [60] and the rheological effects of flaxseed mucilage [49]. CNC and OC muffins revealed relatively stable matrices with intermediate pore sizes, consistent with their higher elastic moduli (*G*′) supporting network reinforcement during baking. AC also formed a moderately cohesive structure, though with larger pores, indicating weaker stabilization compared to CNC or OC.

The SEM observations confirm that the type of milk significantly influenced the organization of the muffin matrix, in line with rheological and textural results. Plant-based milks rich in fibers, mucilage, or phenolic compounds tended to alter pore morphology, producing either compact and dense networks or irregular and expanded pores.

### 3.4. Chemical Composition of Muffin Samples

The chemical composition of muffins is a decisive factor for both nutritional quality and technological functionality. Moisture content affects textural softness and shelf-life stability, while protein and fat levels determine structural integrity and flavor development during baking. Ash and mineral contents provide insights into the micronutrient contribution of different formulations, whereas dietary fiber and total carbohydrate fractions are directly linked with health-promoting properties and caloric density [74].

According to the results presented in Table 4, the control muffin (CC) containing cow’s milk showed the highest protein (8.51 g/100 g) and fat (22.21 g/100 g) contents, which translated into the highest calculated energy value (448.90 kcal/100 g). In contrast, all plant-based formulations exhibited reduced protein levels, ranging from 5.40 to 5.92 g/100 g, with concomitant decreases in fat. Among them, AC and QC muffins displayed the lowest fat contents (19.92 and 19.61 g/100 g, respectively), which led to significantly lower energy values compared to the control (*p* < 0.05). SC and HC muffins stood out with higher dietary fiber (1.02 and 0.99 g/100 g, respectively), highlighting the fiber-enriching potential of these plant-based milks.

**Table 4:** Chemical composition (g/100 g) and mineral composition (mg/1000 g) of muffin samples.

	Moisture	Ash	Protein	Fat	TDF	Total Carbohydrate	Energy (kcal/100 g)
CC	14.45 ± 0.00 ^c^	0.73 ± 0.03 ^bc^	8.51 ± 0.07 ^a^	22.21 ± 0.02 ^a^	0.70 ± 0.01 ^bc^	53.45 ± 0.07 ^e^	448.90 ± 0.00 ^a^
SC	14.91 ± 0.08 ^b^	0.82 ± 0.02 ^b^	5.75 ± 0.07 ^bc^	20.36 ± 0.04 ^c^	1.02 ± 0.02 ^a^	57.20 ± 0.14 ^d^	436.90 ± 0.00 ^f^
HC	14.45 ± 0.03 ^c^	0.73 ± 0.03 ^bc^	5.42 ± 0.04 ^d^	20.36 ± 0.05 ^c^	0.99 ± 0.01 ^a^	58.10 ± 0.00 ^bc^	439.10 ± 0.28 ^d^
WC	14.86 ± 0.07 ^b^	0.72 ± 0.02 ^c^	5.92 ± 0.09 ^b^	20.82 ± 0.06 ^b^	0.49 ± 0.01 ^e^	57.20 ± 0.00 ^d^	440.70 ± 0.14 ^c^
QC	16.18 ± 0.06 ^a^	0.97 ± 0.03 ^a^	5.46 ± 0.00 ^d^	19.61 ± 0.01 ^e^	0.64 ± 0.01 ^cd^	57.10 ± 0.00 ^d^	428.20 ± 0.14 ^g^
FC	14.62 ± 0.02 ^c^	0.82 ± 0.02 ^a^	5.40 ± 0.00 ^d^	20.23 ± 0.03 ^c^	0.60 ± 0.02 ^d^	58.25 ± 0.07 ^b^	437.85 ± 0.35 ^e^
CNC	14.82 ± 0.02 ^b^	0.80 ± 0.01 ^bc^	5.68 ± 0.04 ^c^	20.06 ± 0.02 ^d^	0.73 ± 0.00 ^b^	57.95 ± 0.07 ^c^	436.35 ± 0.07 ^f^
OC	14.20 ± 0.00 ^d^	0.93 ± 0.02 ^a^	5.44 ± 0.00 ^d^	20.76 ± 0.02 ^b^	0.60 ± 0.02 ^d^	58.05 ± 0.07 ^bc^	442.10 ± 0.00 ^b^
AC	13.72 ± 0.05 ^e^	0.76 ± 0.01 ^bc^	5.66 ± 0.01 ^c^	19.92 ± 0.04 ^d^	0.99 ± 0.01 ^a^	58.95 ± 0.07 ^a^	439.75 ± 0.07 ^d^
	**P**		**Na**		**Mg**	**K**	**Ca**
CC	2188.36 ± 103.85 ^a^		2201.69 ± 121.29 ^a^		138.99 ± 9.98 ^b^	1014.14 ± 25.31 ^d^	301.99 ± 13.85 ^a^
SC	1917.80 ± 22.01 ^b^		2015.84 ± 36.11 ^ab^		174.31 ± 1.80 ^a^	1331.41 ± 5.28 ^a^	235.61 ± 3.92 ^bc^
HC	1809.51 ± 53.03 ^bc^		1936.65 ± 58.66 ^bc^		171.40 ± 4.73 ^a^	1163.73 ± 33.21 ^bc^	252.08 ± 2.63 ^b^
WC	1669.66 ± 39.61 ^c^		1802.11 ± 51.70 ^c^		168.99 ± 4.29 ^a^	1142.05 ± 39.47 ^bc^	155.31 ± 0.79 ^d^
QC	1765.32 ± 21.23 ^bc^		1896.96 ± 27.27 ^bc^		171.22 ± 0.98 ^a^	1153.24 ± 14.43 ^bc^	146.15 ± 3.06 ^d^
FC	1698.42 ± 13.60 ^c^		1776.845 ± 11.96 ^c^		169.43 ± 0.96 ^a^	1212.24 ± 5.84 ^b^	165.94 ± 1.05 ^d^
CNC	1815.14 ± 8.13 ^bc^		2030.32 ± 17.317 ^ab^		168.96 ± 3.83 ^a^	1141.18 ± 11.53 ^bc^	230.29 ± 1.03 ^c^
OC	1648.82 ± 17.88 ^c^		1769.86 ± 18.17 ^c^		163.69 ± 1.56 ^a^	1120.66 ± 4.24 ^c^	228.54 ± 5.07 ^c^
AC	1721.91 ± 31.98 ^c^		1862.19 ± 33.94 ^bc^		161.80 ± 5.03 ^a^	1169.46 ± 15.75 ^bc^	244.99 ± 0.67 ^bc^

Results are displayed as the means ± standard deviation. Means followed by the different letter within a columns are significantly different (*p* < 0.05 CC: Control muffin, SC: Soy milk muffin, HC: Hazelnut milk muffin, WC: Walnut milk muffin, QC: Quinoa milk muffin, FC: Flaxseed milk muffin, CNC: Coconut milk muffin, OC: Oat milk muffin, AC: Almond milk muffin.

The mineral profile also varied markedly among samples. Control muffins were richer in calcium (301.99 mg/100 g) compared with the plant-based counterparts, which ranged from 146.15 mg/100 g in QC to 252.08 mg/100 g in HC. On the other hand, SC muffin contained the highest potassium content (1331.41 mg/100 g), nearly 30% higher than the control. CC, SC and CNC muffins showed high sodium concentrations (>2000 mg/100 g), exceeding the WHO recommended daily maximum sodium intake of 2000 mg [75], whereas the other formulations remained between 1770 and 1937 mg/100 g. Magnesium content was consistently higher in plant-based samples (161.8–174.3 mg/100 g) than in the control (138.99 mg/100 g), pointing to their potential in enhancing micronutrient diversity.

The compositional shifts observed here reflect well-known patterns in dairy substitution. Cow’s milk typically contributes higher protein and calcium levels, while plant-based milks reduce energy density and fat content but increase fiber and magnesium [76,77]. Soy- and nut-based systems are particularly associated with improved fiber fractions in bakery applications [78], whereas reduced calcium in quinoa and walnut milks mirrors earlier findings on mineral dilution in gluten-free formulations [77]. Elevated sodium in SC and CNC muffins (>2000 mg/100 g) aligns with reports that some commercial plant milks contain added salts for stabilization [79]. Such differences highlight the dual challenge and opportunity of reformulation: achieving nutritional adequacy while maintaining functional properties.

### 3.5. Fatty Acid Composition

The fatty acid composition of bakery products plays a decisive role in both nutritional quality and technological functionality. Saturated fatty acids (SFA) are generally associated with structural stability but excessive intake is linked with cardiovascular diseases, while unsaturated fatty acids (UFA), including monounsaturated (MUFA) and polyunsaturated fatty acids (PUFA), are considered beneficial for lipid metabolism and inflammation regulation [80]. In muffins, the choice of milk replacer can alter the lipid profile by modulating the proportion of these fatty acid groups.

The results given in Table 5 reveal that palmitic acid (C16:0) and stearic acid (C18:0) were the dominant SFAs across all samples. CC muffin showed significantly higher SFA content (13.40%) compared to plant-based formulations, which ranged between 11.44 and 11.98%. This demonstrates that substitution with alternative milks successfully reduced the proportion of SFAs, thus potentially improving the nutritional lipid quality. Among plant-based milks, FC and QC muffins exhibited slightly elevated SFA values relative to other vegan samples. Although both flaxseed and quinoa are generally recognized for their high PUFA content, flaxseed also contains appreciable amounts of palmitic and stearic acids [81], while quinoa oil includes approximately 20% palmitic acid alongside oleic and linoleic acids [82]. These intrinsic profiles likely explain the modest increase in SFA observed in FC and QC formulations.

Oleic acid (C18:1) was the predominant MUFA, with values consistently around 35% in all formulations, showing no significant differences (*p* > 0.05). This uniformity indicates that the replacement of cow’s milk with plant-based alternatives did not markedly affect MUFA content, which is favorable given the well-known role of oleic acid in supporting cardiovascular health [83]. Linoleic acid (C18:2) represented the main PUFA, ranging from 50.33% in CC to 53.21% in WC. Muffins formulated with walnut, soy, and oat milk exhibited the highest PUFA levels, in agreement with the intrinsic fatty acid profiles of these plant sources, which have been reported to contain higher proportions of unsaturated fatty acids compared to dairy counterparts [84]. Conversely, flaxseed and coconut milk muffins showed comparatively lower PUFA proportions, despite flaxseed being widely recognized as a rich source of α-linolenic acid (C18:3) [85]. Notably, WC and FC muffins recorded the highest α-linolenic acid contents (0.341% and 0.309%, respectively), confirming their potential as functional formulations enriched in omega-3 fatty acids [86].

Reviews emphasize that bovine milk fat is naturally enriched in palmitic and stearic acids, which play a role in gas retention and structural stabilization of baked goods. However, plant-based substitutes introduce higher proportions of oleic and linoleic acids, which enhance nutritional quality and provide additional health benefits [13]. Thus, the observed increase in UFA content, particularly in walnut- and flaxseed-based muffins, indicates a clear advantage from both technological and nutritional perspectives. UFAs accounted for 86–88% of the lipid fraction in all formulations, with the highest TUFA values recorded in plant-based muffins (>88%). This shift towards a more favorable unsaturated-to-saturated fatty acid ratio underscores the potential of plant-based milks to improve the health profile of muffins without compromising structural integrity. These findings confirm that strategic use of milk alternatives can deliver bakery products with both functional performance and enhanced nutritional value.

### 3.6. Total Phenolic Content and Antioxidant Capacity and In Vitro Bioaccesibility

#### 3.6.1. Plant-Based Milks

The antioxidant properties of the milk samples, evaluated by TPC, CUPRAC, and DPPH assays across simulated digestion phases, revealed pronounced differences among sources (Table 6). Phenolic compounds are key contributors to the functional potential of plant-based milks, and their stability and release during gastrointestinal transit determine the extent of physiological benefits [87].

In terms of TPC, cow’s milk (CM) exhibited the highest undigested value (7.876 mg GAE/g). However, digestion markedly reduced its phenolic contribution, yielding a bioaccessibility index of only 25.5%. In contrast, several plant-based alternatives showed modest initial TPC but markedly higher release upon digestion. Coconut milk (CNM) and hazelnut milk (HM) reached bioaccessibility indices of 1663% and 617%, respectively, indicating efficient liberation of bound phenolics. A similar trend was reported in hazelnut-based plant beverages, where in vitro digestion markedly enhanced phenolic release [88]. Quinoa (QM) milks also demonstrated enhanced post-digestive values, with bioaccessibility exceeding 200%. These outcomes highlight that although dairy milk retains a rich starting profile, plant-based milks often outperform it in terms of actual phenolic availability after digestion. A similar observation was made in fortified hazelnut-based plant beverages, where in vitro digestion substantially enhanced phenolic release [88].

CUPRAC activity followed a similar trend. WM exhibited the highest intestinal CUPRAC (1.693 µmol TE/g), while CNM stood out with the greatest relative bioaccessibility (1700%) due to its initially low values. FM and QM also demonstrated substantial increases (213% and 786%, respectively), whereas CM declined markedly to 45.7%. These findings may indicate that both absolute antioxidant capacity and relative bioaccessibility are strongly shaped by the milk matrix, with lipid-rich or fiber–protein–associated systems particularly effective in stabilizing or releasing redox-active compounds during digestion [89].

DPPH radical scavenging activity exhibited pronounced variability among milk types. SM, HM, CNM, and OM reached the highest intestinal values (2.26, 2.25, 2.23, and 2.21 µmol TE/g, respectively), while CM declined sharply to 0.35 µmol TE/g. Despite the lower activities of several plant-based samples, their relative bioaccessibility indices exceeded that of CM, particularly in OM (27.8%) and AM (11.2%). These findings emphasize that the botanical composition of plant-based milks determines both antioxidant release and stability under gastrointestinal conditions.

In most plant-based milks, DPPH values were higher than CUPRAC after digestion, reflecting the strong radical-scavenging activity of phenolic compounds such as flavonoids and phenolic acids. By contrast, cow’s and walnut milk showed higher CUPRAC values, which is consistent with the presence of lipid-associated antioxidants better captured by reducing-power assays [90].

#### 3.6.2. Muffins

The antioxidant properties of the muffin samples, assessed by TPC, CUPRAC, and DPPH across simulated digestion phases, are summarized in Table 7. Digestion significantly altered the levels of phenolic compounds. These changes demonstrate the complex interactions of proteins, starches, and lipids with polyphenols, beginning during baking and continuing throughout gastrointestinal breakdown [91].

In the undigested state, the highest total phenolic content (TPC) was found in CNC and QC (3.59 and 2.88 mg GAE/g), followed by OC and CC (2.48 and 2.08 mg GAE/g). The elevated TPC in CNC can be linked to phenolic compounds naturally occurring in coconut milk, while the higher values in OC are attributed to avenanthramides, the characteristic phenolic alkaloids of oats with strong antioxidant activity [92,93]. After digestion, TPC bioaccessibility showed strong variation. FC and WC displayed the highest increases (299.7% and 161.3%, respectively), pointing to efficient release of bound polyphenols. CC reached moderate values (168.2%), while CNC and OC showed much lower bioaccessibility (65.8% and 13.6%), likely due to their specific compositional matrices enhancing the release of bound phenolics during digestion, whereas the limited release in CNC and OC highlights the strong matrix–polyphenol interactions that may hinder phenolic availability [89].

CUPRAC activity also varied widely among formulations. The highest intestinal CUPRAC values were observed in AC (0.89 µmol TE/g) and CC (0.63 µmol TE/g) while SC and WC exhibited moderate values (0.52 and 0.47 µmol TE/g, respectively). Bioaccessibility percentages were high in AC (44,400%), CNC (993.6%) and SC (645%). Matrix composition strongly influences the fate of phenolics during digestion. Lipid- and protein-rich systems, such as those derived from nuts or coconut, can protect phenolics through micellization or protein complexation, thereby enhancing their stability and release. By contrast, polysaccharide-rich matrices in soy and cereals tend to capture phenolics within their structures, reducing their accessibility for absorption [94,95].

DPPH radical scavenging activity also presented variability. SC and OC showed the highest intestinal values (1.85 and 1.82 µmol TE/g, respectively), whereas CC exhibited the lowest intestinal value (1.58 µmol TE/g). This pattern indicates that gastrointestinal conditions strongly modulate radical scavenging responses, with some formulations retaining or even enhancing activity while others exhibit noticeable losses. The relatively stable activity of SC and OC may be linked to phenolic structures with greater resistance to enzymatic and pH-induced degradation, allowing more sustained radical scavenging capacity. This suggests that the digestive environment, particularly the transition from acidic gastric to neutral intestinal conditions, is a critical determinant of phenolic antioxidant stability [96]. Consistent with this, investigations on bakery systems have also shown that the stability of phenolics during gastrointestinal passage is highly matrix-dependent, with certain plant-based formulations maintaining higher antioxidant activity after digestion compared to dairy-based counterparts [15].

The results show that replacing cow’s milk with plant-based alternatives changes the antioxidant potential of muffins by affecting both activity levels and post-digestive bioaccessibility. Seed- and pseudocereal-based milks such as flaxseed, quinoa, and coconut gave the highest improvements, while almond and oat led to the weakest outcomes due to limited phenolic release. Plant-based milks influence antioxidant responses and make certain formulations promising functional bakery products with stronger post-digestive bioactivity.

### 3.7. Total Amino Acid (AA) Composition

The amino acid composition of muffins, presented in Table 8, revealed distinct variations among the control and plant-based formulations. Beyond their structural role in proteins, amino acids participate in a wide range of physiological processes including energy metabolism, immune response, neurotransmission, and tissue repair [97].

Acidic amino acids were dominant in WC, QC, and FC muffins. WC (1.895 g/100 g) and QC (1.850 g/100 g) showed glutamic acid levels nearly fourfold higher than CC (0.476 g/100 g), while QC also contained the highest aspartic acid (0.224 g/100 g vs. 0.065 g/100 g in CC). These increments indicate that certain plant-based alternatives, particularly walnut and quinoa, enhanced the abundance of flavor-active amino acids, consistent with their reported contribution to umami perception [98,99].

Among the basic amino acids, arginine was most abundant. WC (1.928 g/100 g) recorded nearly double the CC level (1.007 g/100 g), followed by QC (1.780 g/100 g) and FC (1.398 g/100 g). This highlights the arginine-rich nature of nut- and seed-derived proteins, which is nutritionally relevant since arginine is involved in nitric oxide synthesis and immune regulation [100]. Lysine content varied substantially among samples. While CC contained 0.114 g/100 g, levels dropped markedly in SC (0.010 g/100 g) and HC (0.006 g/100 g), supporting the well-known limitation of lysine in many plant proteins [101]. However, certain formulations such as WC (0.246 g/100 g) and QC (0.228 g/100 g) showed elevated lysine contents, indicating that specific plant sources may partially compensate for this deficiency.

Neutral amino acids also showed strong variability. WC and QC demonstrated the highest values for several essential neutral amino acids, including leucine (0.602 and 0.589 g/100 g, respectively), nearly doubling CC (0.330 g/100 g). FC also showed an elevated leucine level (0.427 g/100 g). By contrast, SC and HC contained very low leucine (0.051 and 0.031 g/100 g). Similar patterns were observed for valine and isoleucine, which peaked in WC and QC but were minimal in soy- and hazelnut-based formulations. These branched-chain amino acids (BCAAs) are fundamental to protein synthesis and energy metabolism, and their variability has direct implications for the nutritional quality of vegan muffins [102]. Methionine also displayed a similar trend: while WC and QC maintained levels comparable to CC, SC and HC recorded substantial reductions (0.015 and 0.013 g/100 g, respectively, vs. 0.088 g/100 g in CC). This underscores methionine as another limiting amino acid in soy and nut-based milk alternatives. A distinctive feature was the presence of hydroxyproline in WC (0.225 g/100 g), QC (0.200 g/100 g), and OC (0.078 g/100 g), whereas it was undetectable in CC. Hydroxyproline is generally associated with collagen; its detection in vegan samples may derive from hydrolyzed plant proteins or collagen-like analogues, potentially influencing textural attributes of the muffins [103].

Substituting cow’s milk with plant-based alternatives reshaped the amino acid spectrum of muffins. While CC retained higher methionine, certain formulations such as WC and QC reached lysine levels comparable to or exceeding the control. Moreover, WC, QC, and FC exhibited elevated glutamic acid, arginine, and leucine. These findings underscore both the nutritional limitations and the functional advantages of plant-based milks, emphasizing that amino acid balance in vegan muffins is determined by the specific substitute employed.

### 3.8. Shelf-Life Evaluation of Muffin Samples

During the 30-day storage period, significant differences were observed among the muffin formulations with respect to physicochemical, textural, color, and microbiological properties (Supplementary Tables S1 and S2).

Moisture content showed a biphasic trend. Between day 0 and day 15, moisture levels increased in all samples, due to equilibration and redistribution of water within the matrix. The highest values were recorded in HC and QC (19.94% and 19.87%, respectively), whereas CC and AC remained comparatively lower (17.02% and 18.28%). By day 30, however, moisture declined sharply across all formulations, most prominently in SC (9.17%) and CC (10.07%), while FC retained the highest level (15.71%). This reduction indicates progressive dehydration during storage, a typical phenomenon in bakery systems. The relatively stable hydration in FC may be linked to flaxseed mucilage, which provides water retention capacity [104]. Ash content exhibited moderate variations. On day 30, OC and AC recorded the highest values (1.18% and 1.11%), possibly reflecting concentration effects due to moisture loss and localized redistribution of minerals. In contrast, SC showed the lowest ash value (0.57%). Fat content also declined over time, consistent with lipid oxidation and hydrolysis. The decrease was especially marked in CC (22.21% to 16.66%), whereas WC and SC maintained higher levels (21.84% and 21.73% at day 30). These results reflect the susceptibility of unsaturated fatty acids to oxidation under ambient storage [105].

Textural attributes could be assessed up to day 15, but the same parameters were not applicable at day 30, so the values were not measurable. Hardness increased substantially, particularly in CNC (from 1416.21 gf to 4153.08 gf) and AC (1404.69 gf to 4116.30 gf). A parallel rise in chewiness was observed in AC (748.21 to 1236.76), while springiness and resilience values decreased, indicating structural weakening. Cohesiveness also declined consistently reflecting starch retrogradation and protein–starch interactions that limit crumb elasticity [15].

Color parameters were markedly affected by storage. Crust L* values declined initially, most clearly in CC (58.98 to 54.13), whereas QC maintained higher brightness. Crust a* values remained consistently higher in CC (12.48–14.10), reflecting stronger Maillard browning promoted by egg proteins and lactose [101]. Crust b* values were highest and increased in CC (30.99 to 33.28), but remained lower in WC (24.57 to 23.60). For crumb color, L* decreased notably in OC (70.62 to 62.33), a* values rose in WC (1.22 to 2.78), and b* values declined slightly across most samples. These changes highlight the combined effects of Maillard reactions and plant-derived pigments on color stability during storage.

Aerobic plate counts (APC) varied between 1.5 × 10^2^ CFU/g (OC, day 0) and 9.2 × 10^3^ CFU/g (SC, day 15), remaining below levels typically linked to spoilage. All samples also met the food safety requirements for ready-to-eat bakery products defined by EC No. 2073/2005, as *E. coli*, coliforms, *Staphylococcus aureus*, yeasts, molds, and *Salmonella* spp. were not detected. These results confirm that hygienic conditions were maintained during production and that microbial safety was preserved throughout storage. Importantly, the use of different plant-based milks did not compromise microbiological quality, and all muffin formulations remained stable and safe for 30 days under ambient conditions.

All muffin formulations maintained acceptable quality and microbial stability during 30 days of storage. The main challenges were moisture loss and texture hardening, most pronounced in SC and CC. By contrast, flaxseed- and nut-based variants (FC, AC, CNC) held water better and retained their structure more effectively. Importantly, every formulation remained microbiologically safe under ambient conditions, showing that a wide range of plant-based milks can be used to produce bakery products with an extended shelf life.

### 3.9. Sensorial Characteristics

**Figure 5:**
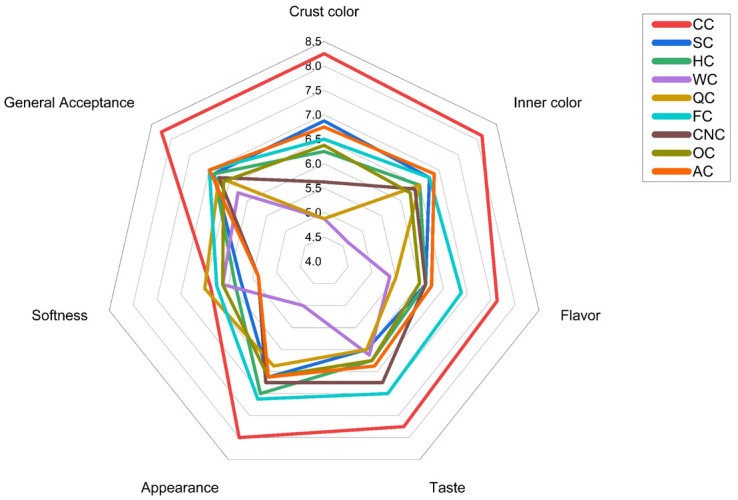
Sensory evaluation profiles of muffin formulations presented as radar plots. CC: Control muffin, SC: Soy milk muffin, HC: Hazelnut milk muffin, WC: Walnut milk muffin, QC: Quinoa milk muffin, FC: Flaxseed milk muffin, CNC: Coconut milk muffin, OC: Oat milk muffin, AC: Almond milk muffin.

Sensory evaluation is a key determinant of consumer acceptance in food products. The results of the panel test (n = 20) are presented in the radar diagram (Figure 5).

Crust and crumb color scores ranged from approximately 5.0 to 8.5, with the control muffins (CC) rated highest, reflecting the contribution of egg proteins and lactose to Maillard browning. The color of baked products is shaped both by the natural pigments of the ingredients and by the changes that occur through their interactions during baking [15]. Flavor and taste values varied between 5.8 and 8.0. CC had the highest taste score as 8.0, while AC (~6.5), FC (~7.0), and CNC (~7.0) also received favorable ratings. In contrast, WC and QC scored lower (~5.8–6.0), likely due to their darker crumb and more pronounced flavor notes. Softness scores were below 6.0 across all formulations. A clear correlation was observed between the rheological and sensory findings. Muffins prepared with plant-based milks exhibiting lower storage modulus (G′) and higher loss tangent (tan *δ*) values were perceived as softer and moister by the sensory panel. This indicates that the viscoelastic behavior of muffin batters directly influences perceived tenderness and moistness, emphasizing that plant-based milks can modulate batter structure to achieve desirable sensory textures and overall acceptance in vegan bakery products. General acceptance ranged between 6.0 and 8.5, with CC most preferred (~8.5), followed closely by AC and FC, while WC remained lowest (~6.0). Despite these differences, all muffins scored above the sensory acceptability threshold, indicating that consumer acceptance was broadly maintained. Similar trends have been reported in plant-based bakery studies, where nut-based formulations such as almond-enriched muffins achieved overall acceptability scores above 7.0 on a 9-point hedonic scale [15], while seed-based formulations such as flaxseed-enriched muffins maintained overall acceptance within the 6–7 range, although flavor and texture attributes were modified [106].

### 3.10. Chemometric Analysis

#### 3.10.1. Correlation Coefficient

**Figure 6:**
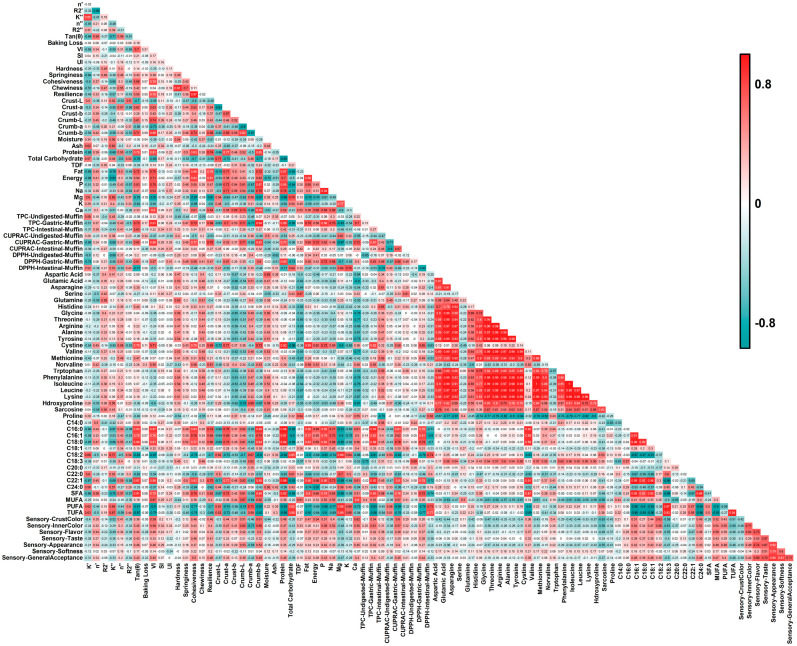
Pearson correlation heatmap of physicochemical, compositional, textural, and functional attributes of the samples.

The Pearson correlation heatmap (Figure 6) provided an integrative overview of interrelationships among rheological model parameters, physicochemical attributes, compositional variables, and functional indices of aquafaba-based vegan muffins. Several strong positive and negative correlations were observed, reflecting how rheological behavior, moisture dynamics, and antioxidant potential collectively define product quality.

A strong positive correlation between the storage and loss consistency indices (K′–K″, R2 = 0.85) indicated that samples with higher elastic consistency also exhibited greater viscous response, reflecting coherent viscoelastic network formation in batters stabilized by plant-based hydrocolloids [107]. Conversely, K′ correlated negatively with DPPH values in undigested and gastric phases but positively in the intestinal phase (−0.71 ≤ R^2^ ≤ 0.77), suggesting that matrix elasticity influenced the release of antioxidant compounds during digestion.

Hardness and chewiness were highly correlated (R^2^ = 0.91), as were resilience and cohesiveness (R^2^ = 0.97), confirming that nutrient-rich formulations supported stronger internal bonding and improved structural recovery. Cohesiveness also correlated positively with protein and fat contents (R^2^ ≈ 0.85), highlighting the reinforcing role of macronutrients in texture development. Similarly, the volume index showed positive associations with protein and TPC in the gastric phase (R^2^ = 0.81–0.91), linking increased volume with enhanced phenolic release and color intensity (crumb b*, R^2^ = 0.88).

Strong intercorrelations among amino acids such as aspartic acid, glutamic acid, and glycine (R^2^ = 0.87–0.90) reflected their shared origin in legume- and seed-derived proteins. Proline, in contrast, exhibited a pronounced negative correlation with these amino acids (R^2^ ≈ –0.80), suggesting that its cyclic structure limited participation in hydrogen-bonded protein networks [108]. The antioxidant assays also displayed tight relationships: TPC and CUPRAC values across digestive phases were strongly aligned (R^2^ ≥ 0.90), demonstrating coherent redox behavior of polyphenol-rich systems [109].

In addition to the compositional and rheological relationships, strong associations were observed among the sensory attributes themselves, as well as between instrumental and perceptual measures. General acceptability was closely linked with softness, appearance, and taste (R^2^ = 0.72–0.86), confirming that consumer preference was primarily driven by mouthfeel and visual quality. Instrumental cohesiveness and resilience also correlated positively with sensorial general acceptability (R^2^ ≈ 0.45–0.50), indicating that structurally stable matrices were perceived as softer and more pleasant. Positive correlations between crumb L* and b* values and sensory color scores (R^2^ = 0.51–0.66) indicate that lighter and more yellowish crumb tones were perceived as visually appealing, while the negative association between crumb a* and sensory inner color (R^2^ = –0.71) suggests that excessive reddish hues were less favored. Conversely, the weak negative relationship between crust L* and crust color perception (R^2^ ≈ −0.30) implies that a slightly darker crust was preferred for an optimal baked appearance

The results indicate that rheological strength, compositional density, and antioxidant capacity are the primary factors shaping the physical and functional characteristics of plant-based muffins, forming the statistical basis for the PCA and HCA results discussed in the following sections.

#### 3.10.2. Principal Component Analysis

**Figure 7:**
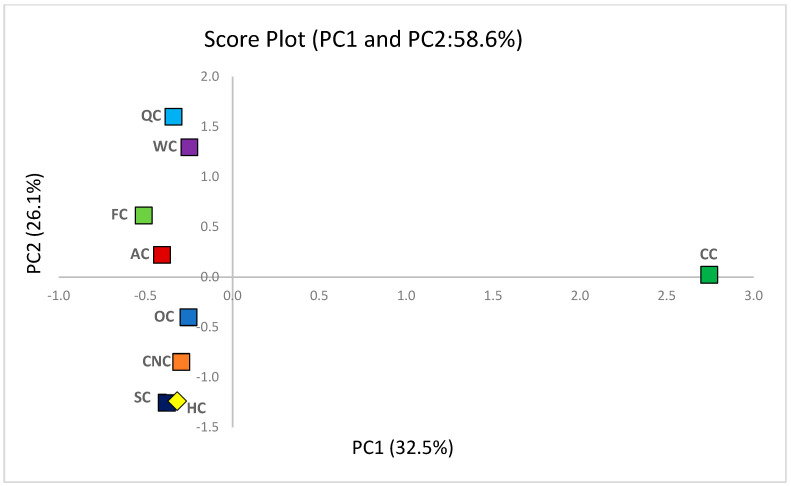
PCA score plot of samples. CC: Control muffin, SC: Soy milk muffin, HC: Hazelnut milk muffin, WC: Walnut milk muffin, QC: Quinoa milk muffin, FC: Flaxseed milk muffin, CNC: Coconut milk muffin, OC: Oat milk muffin, AC: Almond milk muffin.

**Figure 8:**
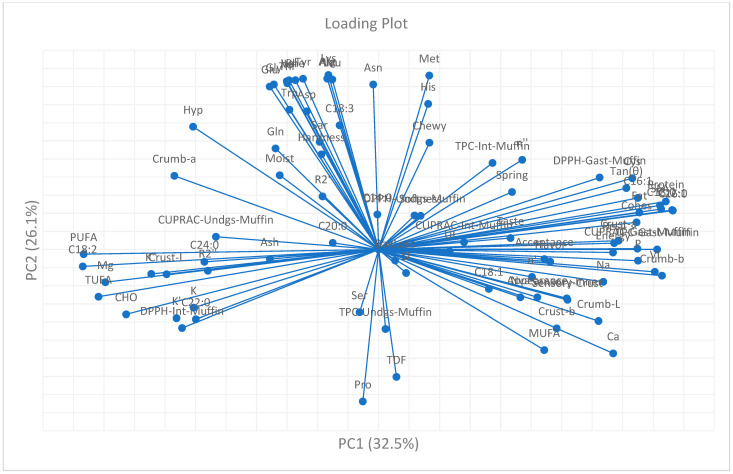
PCA loading plot of samples.

PCA was performed to reduce the multidimensional dataset and to identify the principal factors governing the physical, compositional, and functional variations among aquafaba-based vegan muffins (Supplementary Table S3). The first two principal components (PC1 and PC2) explained 58.6% of the total variance (Figure 7 and Figure 8), indicating an adequate dimensional reduction to visualize the dominant patterns within the dataset. PC1 (32.5% of the variance) was primarily defined by rheological and compositional attributes, whereas PC2 (26.1%) reflected variations in antioxidant response and amino acid composition.

Positive loadings of protein (0.94), fat (0.86), saturated fatty acids (0.92), and volume index (0.91) on PC1 demonstrated that nutrient-rich and structurally coherent batters exhibited higher viscoelastic strength and greater expansion during baking. These variables were closely aligned with cohesiveness (0.85), resilience (0.77), and crumb b* (0.93), indicating that color development and textural uniformity were directly related to macronutrient content and matrix organization. In contrast, negative loadings of PUFA (−0.97), TUFA (−0.92), and total carbohydrate (−0.83) denoted an opposite compositional trend associated with unsaturated lipid enrichment and carbohydrate dominance, which are typically linked with softer, less elastic matrices [110,111].

PC2 exhibited strong positive loadings for a cluster of amino acids, including arginine (0.94), alanine (0.94), tyrosine (0.94), valine (0.94), and methionine (0.96), reflecting the influence of proteinogenic components on the nutritional profile of legume- and seed-based formulations. Negative loading of proline (−0.84) on the same axis confirmed its antagonistic behavior due to its cyclic imino structure, which limits participation in hydrogen-bonded protein networks. In parallel, antioxidant indices measured in the gastric phase of the milk fraction—including total phenolic content (TPC: 0.76), CUPRAC (0.73), and DPPH (0.73)—showed co-alignment along PC2, highlighting phase-dependent clustering of polyphenolic responses during simulated digestion.

The PCA score plot revealed clear sample differentiation. The control muffin (CC) was distinctly separated along PC1 due to its higher protein, fat, and SFA loadings, reflecting its strong structural and compositional integrity. Plant-based milk formulations formed subgroups on the negative side of PC1: quinoa (QC) and walnut (WC) muffins positioned higher along PC2, indicating enhanced amino acid and antioxidant characteristics [112,113], while soy (SC) and hazelnut (HC) muffins clustered lower, corresponding to higher unsaturated lipid content and reduced viscoelastic strength [114].

PCA confirmed the multivariate relationships suggested by the correlation analysis. The integrated clustering of rheological, compositional, and antioxidant parameters demonstrates that the structural stability, amino acid complexity, and redox potential jointly determine the functionality and nutritional quality of aquafaba-based vegan muffins.

#### 3.10.3. Hierarchical Cluster Analysis

**Figure 9:**
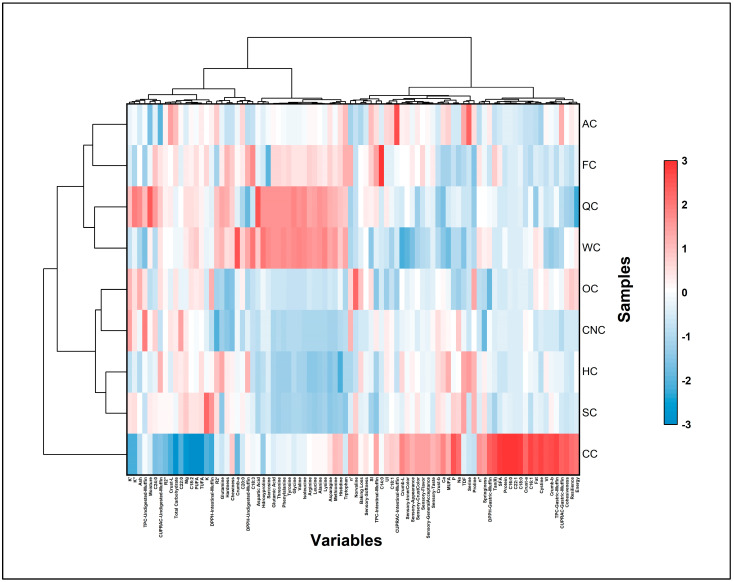
Cluster map of muffin samples.

HCA was performed to evaluate the relationships among muffin formulations and to identify groups with comparable physicochemical and functional characteristics. Clustering was conducted based on Ward’s method with Euclidean distance as the similarity metric. The resulting dendrogram (Figure 9) illustrated the overall relational pattern among samples. This multivariate approach complemented the PCA findings, providing an enhanced interpretation of formulation proximity and differentiation within the dataset [115].

Cluster I was composed of CC, SC, HC, WC, and QC samples, all of which exhibited comparable patterns associated with elevated levels of unsaturated lipids, moderate antioxidant capacity, and limited viscoelastic strength. The alignment of soy and hazelnut muffins within this cluster indicates that lipid-dominant matrices improved moisture retention but weakened gas-cell stability, producing softer and less cohesive textures [114]. Similarly, walnut- and quinoa-based formulations displayed parallel behavior, as their polyunsaturated lipid profiles favored nutritional enhancement but restricted elastic recovery within the baked matrix. These observations suggest that formulations dominated by plant-derived oils formed a coherent group defined by moderate structural integrity and reduced rheological strength, consistent with previous reports on nut- and seed-based bakery systems [57,68].

Cluster II included FC, CNC, OC, and AC muffins, which shared higher levels of protein and dietary fiber, as well as stronger textural resilience and enhanced antioxidant bioaccessibility. The proximity of FC and CNC samples reflected the contribution of mucilage- and hydrocolloid-rich compounds that supported a cohesive and elastic crumb network [43,116]. OC and AC muffins were also positioned closely, characterized by improved viscoelastic balance and elevated phenolic content—an effect likely arising from synergistic starch–protein–fiber interactions within their matrices. These results highlight the capacity of hydrocolloid- and fiber-dominant systems to reinforce both mechanical and biofunctional quality in vegan bakery products [60,68].

Together, the PCA and HCA outcomes demonstrate that the type of plant-based milk exerts a decisive influence on the multidimensional behavior of aquafaba-based muffins. Fiber- and hydrocolloid-rich milks (Cluster II) promoted structural cohesion and functional enhancement, whereas lipid-oriented formulations (Cluster I) were associated with softer, less elastic textures. This integrated interpretation underscores the relevance of multivariate modeling for identifying formulation-specific relationships in plant-based bakery systems.

## 4. Conclusions

This study provides an integrative understanding of how eight different plant-based milks influence the structural, functional, and nutritional characteristics of muffins, contributing new insights into dairy replacement strategies for bakery products. Among the alternatives, quinoa, flaxseed, and coconut milks emerged as the most promising in enhancing viscoelastic stability and antioxidant potential, while oat and almond formulations exhibited weaker rheological and bioactive performance. The results showed that milk substitution did not impair baking performance or consumer acceptability, confirming the technological feasibility of dairy replacement in muffin production. Distinct color and compositional variations were linked to the inherent pigment and macronutrient profiles of each milk matrix, reflecting ingredient-specific functionality.

This work fills a research gap by systematically evaluating the influence of plant-based milks on rheology, amino acid balance, phenolic content, and sensory quality in a unified framework. The integration of multivariate analyses (PCA and HCA) provided a holistic understanding of sample clustering, confirming that compositional traits govern both mechanical and bioactive behavior. Collectively, the findings emphasize that non-dairy milks can diversify the nutritional and functional landscape of bakery products while maintaining desirable physical and sensory quality.

From an application standpoint, the results support the use of quinoa-, flaxseed-, and coconut-based milks as functional dairy alternatives for clean-label and vegan bakery systems. Their ability to combine acceptable texture, balanced amino acid composition, and enhanced antioxidant capacity presents a valuable basis for industrial-scale formulation. These findings demonstrate strong potential for commercial implementation in the plant-based bakery sector, offering sustainable and allergen-free options for product innovation. Recognizing the distinct molecular contributions of each plant matrix is critical for optimizing structure–function relationships in non-dairy baked goods.

This study has certain limitations that should be acknowledged. The use of a single source of flour and fat may restrict the generalizability of the findings, as variations in ingredient origin and composition could affect muffin rheology, texture, and sensory perception. Moreover, the evaluation of storage stability was limited to 30 days, which may not fully capture long-term textural or oxidative changes. Future research should therefore include multiple ingredient sources, diverse fat compositions, and extended storage durations to validate and expand the applicability of the present results, as well as to investigate the shelf-life and stability of plant-based milk muffins under varying environmental conditions. In addition, scaling-up studies and cost–benefit analyses would be valuable to assess the feasibility of industrial production, while expanded sensory panels across diverse consumer populations could support market-oriented formulation optimization. Comprehensive analyses of volatile aroma profiles, phenolic transformations during baking, and in vivo antioxidant bioavailability would further substantiate the health-related claims of such products. Moreover, conducting extensive consumer hedonic tests and optimizing formulations to accommodate gluten-free and other dietary preferences would enhance product inclusivity. Establishing standardized quality parameters for plant-based milks used in vegan muffin production is also recommended, as differences arising from their manufacturing processes may substantially affect final product characteristics. Finally, exploring hybrid formulations that combine two or more milk alternatives could uncover synergistic effects in texture, flavor, and nutritional value, supporting the development of next-generation functional bakery products.

## Figures and Tables

**Table 1:** Ingredient composition of muffin formulations.

Ingredients (g)	CC	SC	HC	WC	QC	FC	CNC	OC	AC
Wheat flour	200 g	200 g	200 g	200 g	200 g	200 g	200 g	200 g	200 g
Sunflower oil	100 g	100 g	100 g	100 g	100 g	100 g	100 g	100 g	100 g
Whole egg	90 g	0	0	0	0	0	0	0	0
Aquafaba (chickpea)	0	90 g	90 g	90 g	90 g	90 g	90 g	90 g	90 g
Sugar	150 g	150 g	150 g	150 g	150 g	150 g	150 g	150 g	150 g
Cow milk	32 g	0	0	0	0	0	0	0	0
Soy milk	0	32 g	0	0	0	0	0	0	0
Hazelnut milk	0	0	32 g	0	0	0	0	0	0
Walnut milk	0	0	0	32 g	0	0	0	0	0
Quinoa milk	0	0	0	0	32 g	0	0	0	0
Flaxseed milk	0	0	0	0	0	32 g	0	0	0
Coconut milk	0	0	0	0	0	0	32 g	0	0
Oat milk	0	0	0	0	0	0	0	32 g	0
Almond milk	0	0	0	0	0	0	0	0	32 g
Baking powder	5 g	5 g	5 g	5 g	5 g	5 g	5 g	5 g	5 g
Vanillin	3 g	3 g	3 g	3 g	3 g	3 g	3 g	3 g	3 g

Ingredients at 21 ± 1 °C; CC: Control muffin, SC: Soy milk muffin, HC: Hazelnut milk muffin, WC: Walnut milk muffin, QC: Quinoa milk muffin, FC: Flaxseed milk muffin, CNC: Coconut milk muffin, OC: Oat milk muffin, AC: Almond milk muffin.

**Table 2:** Dynamic shear parameters of power-law functions describing the *G*′ and *G*″ values of muffin samples.

	$G^{\prime }=K^{\prime }{\left(\omega \right)}^{n^{\prime }}$			$G^{\prime \prime }=K^{\prime \prime }{\left(\omega \right)}^{n^{\prime \prime }}$			tan *δ*
Samples	*K*′ (Pa.s^n^)	*n*′	*R* ^2^	*K*″ (Pa.s^n^)	*n*″	*R* ^2^	
CC	598.32 ± 17.49 ^g^	0.458 ± 0.03 ^ab^	0.942	532.25 ± 23.31 ^f^	0.533 ± 0.12 ^a^	0.937	0.778 ± 0.02 ^a^
SC	1524.80 ± 23.53 ^c^	0.342 ± 0.02 ^c^	0.983	1099.00 ± 13.62 ^b^	0.426 ± 0.03 ^c^	0.938	0.609 ± 0.01 ^d^
HC	1048.20 ± 35.48 ^f^	0.301 ± 0.05 ^d^	0.997	906.83 ± 18.23 ^d^	0.452 ± 0.02 ^bc^	0.940	0.643 ± 0.00 ^cd^
WC	1076.40 ± 16.25 ^f^	0.300 ± 0.03 ^d^	0.995	933.78 ± 20.18 ^d^	0.455 ± 0.05 ^bc^	0.922	0.636 ± 0.00 ^cd^
QC	1671.60 ± 15.22 ^b^	0.301 ± 0.02 ^d^	0.998	1408.10 ± 22.78 ^a^	0.437 ± 0.04 ^bc^	0.940	0.652 ± 0.00 ^c^
FC	1183.40 ± 22.62 ^e^	0.490 ± 0.04 ^a^	0.908	777.07 ± 20.65 ^e^	0.483 ± 0.09 ^ab^	0.938	0.692 ± 0.01 ^b^
CNC	1838.30 ± 19.13 ^a^	0.460 ± 0.04 ^ab^	0.871	1089.50 ± 12.33 ^b^	0.438 ± 0.03 ^bc^	0.925	0.636 ± 0.02 ^cd^
OC	1706.20 ± 29.52 ^b^	0.423 ± 0.03 ^b^	0.903	1082.70 ± 28.82 ^b^	0.442 ± 0.11 ^bc^	0.934	0.631 ± 0.02 ^cd^
AC	1235.10 ± 29.01 ^d^	0.295 ± 0.05 ^d^	0.989	970.55 ± 15.82 ^c^	0.420 ± 0.14 ^c^	0.959	0.624 ± 0.01 ^cd^

Results are displayed as the means ± standard deviation. Means followed by the different letter within a columns are significantly different (*p* < 0.05). tan *δ*: Loss factor at 1.35 rad s^−1^. CC: Control muffin, SC: Soy milk muffin, HC: Hazelnut milk muffin, WC: Walnut milk muffin, QC: Quinoa milk muffin, FC: Flaxseed milk muffin, CNC: Coconut milk muffin, OC: Oat milk muffin, AC: Almond milk muffin.

**Table 3:** Physical properties of muffin samples.

		CC	SC	HC	WC	QC	FC	CNC	OC	AC
Volumetric properties										
Baking Loss (%)		6.41 ± 0.39 ^a^	6.08 ± 0.39 ^a^	6.67 ± 0.64 ^a^	6.07 ± 1.12 ^a^	6.34 ± 0.89 ^a^	6.31 ± 0.14 ^a^	5.91 ± 0.29 ^a^	6.91 ± 0.64 ^a^	5.63 ± 1.03 ^a^
VI (mm)		119.00 ± 3.61 ^a^	95.67 ± 1.15 ^bcde^	101.33 ± 0.58 ^bc^	91.00 ± 2.65 ^e^	94.67 ± 1.15 ^cde^	94.00 ± 0.00 ^de^	95.33 ± 1.15 ^cde^	102.33 ± 0.58 ^b^	100.67 ± 5.13 ^bcd^
SI (mm)		9.00 ± 1.00 ^a^	7.33 ± 0.58 ^a^	8.67 ± 1.53 ^a^	7.00 ± 1.00 ^a^	9.33 ± 1.53 ^a^	10.00 ± 1.73 ^a^	8.67 ± 0.58 ^a^	9.67 ± 1.15 ^a^	10.33 ± 1.53 ^a^
UI (mm)		1.00 ± 1.00 ^a^	0.00 ± 1.73 ^a^	0.67 ± 0.58 ^a^	−0.33 ± 0.58 ^a^	0.67 ± 0.58 ^a^	0.00 ± 1.00 ^a^	0.67 ± 1.53 ^a^	−1.00 ± 0.00 ^a^	1.00 ± 1.73 ^a^
Texture										
Hardness (gf)		1284.74 ± 104.18 ^bc^	1616.01 ± 205.37 ^a^	1584.69 ± 141.61 ^a^	976.59 ± 68.83 ^d^	1158.40 ± 82.19 ^c^	1015.00 ± 3.33 ^cd^	1416.21 ± 143.37 ^ab^	1459.38 ± 101.54 ^ab^	1404.69 ± 103.67 ^ab^
Springiness		0.91 ± 0.01 ^a^	0.88 ± 0.01 ^b^	0.88 ± 0.02 ^b^	0.86 ± 0.00 ^c^	0.88 ± 0.01 ^b^	0.84 ± 0.01 ^d^	0.89 ± 0.01 ^ab^	0.89 ± 0.01 ^ab^	0.89 ± 0.03 ^ab^
Cohesiveness		0.71 ± 0.00 ^a^	0.58 ± 0.00 ^c^	0.59 ± 0.03 ^c^	0.64 ± 0.02 ^b^	0.62 ± 0.01 ^bc^	0.60 ± 0.01 ^c^	0.60 ± 0.03 ^c^	0.62 ± 0.01 ^bc^	0.60 ± 0.01 ^c^
Chewiness		828.54 ± 70.46 ^a^	823.56 ± 117.80 ^a^	832.87 ± 116.75 ^a^	534.95 ± 23.83 ^d^	633.71 ± 33.72 ^cd^	513.33 ± 9.88 ^d^	755.78 ± 74.46 ^bc^	808.05 ± 64.91 ^ab^	748.21 ± 72.95 ^bc^
Resilience		0.33 ± 0.00 ^a^	0.25 ± 0.00 ^c^	0.26 ± 0.02 ^c^	0.30 ± 0.02 ^ab^	0.29 ± 0.01212 ^b^	0.27 ± 0.01 ^bc^	0.27 ± 0.02 ^bc^	0.28 ± 0.01 ^bc^	0.26 ± 0.01 ^c^
Color										
Crust	L*	58.98 ± 3.05 ^c^	64.65 ± 2.77 ^ab^	62.89 ± 2.19 ^bc^	65.12 ± 1.17 ^ab^	65.27 ± 2.35 ^ab^	64.40 ± 2.17 ^ab^	67.46 ± 2.86 ^ab^	63.67 ± 1.06 ^bc^	69.12 ± 1.96 ^a^
	a*	12.48 ± 1.88 ^a^	6.04 ± 3.42 ^b^	6.44 ± 2.67 ^ab^	4.74 ± 0.79 ^b^	3.91 ± 2.97 ^b^	4.45 ± 3.65 ^b^	4.95 ± 2.91 ^b^	5.03 ± 2.45 ^b^	3.21 ± 1.83 ^b^
	b*	30.99 ± 2.86 ^a^	28.96 ± 0.47 ^ab^	28.75 ± 0.90 ^ab^	24.57 ± 0.92 ^b^	24.49 ± 2.61 ^b^	26.34 ± 2.64 ^b^	27.82 ± 1.85 ^ab^	27.74 ± 1.13 ^ab^	25.92 ± 2.26 ^b^
	*ΔE*	-	13.08 ± 4.22 ^a^	11.56 ± 3.24 ^a^	14.33 ± 0.79 ^a^	15.07 ± 4.16 ^a^	14.03 ± 4.11 ^a^	15.85 ± 3.84 ^a^	13.00 ± 2.38 ^a^	18.20 ± 2.70 ^a^
	C*	33.49 ± 2.01 ^a^	29.72 ± 1.05 ^ab^	29.54 ± 1.27 ^ab^	25.03 ± 1.05 ^b^	24.91 ± 2.92 ^b^	26.85 ± 3.21 ^b^	28.34 ± 2.28 ^b^	28.26 ± 1.49 ^b^	26.16 ± 2.42 ^b^
	*h*°	67.88 ± 4.84 ^b^	78.38 ± 6.36 ^ab^	77.51 ± 4.96 ^ab^	79.12 ± 1.38 ^ab^	76.51 ± 3.22 ^ab^	81.01 ± 6.76 ^a^	80.21 ± 5.27 ^a^	79.87 ± 4.55 ^a^	83.19 ± 3.66 ^a^
Crumb	L*	75.03 ± 0.85 ^a^	69.58 ± 0.55 ^ab^	72.02 ± 1.13 ^b^	64.69 ± 1.16 ^c^	69.38 ± 0.82 ^b^	70.46 ± 0.84 ^ab^	69.98 ± 0.63 ^ab^	70.62 ± 1.82 ^ab^	71.43 ± 1.24 ^ab^
	a*	−2.28 ± 0.29 ^d^	−0.90 ± 0.04 ^bc^	−0.86 ± 0.15 ^bc^	1.22 ± 0.10 ^a^	−1.15 ± 0.19 ^c^	−0.66 ± 0.05 ^b^	−0.99 ± 0.13 ^bc^	−1.02 ± 0.22 ^bc^	−0.99 ± 0.16 ^bc^
	b*	24.33 ± 0.80 ^a^	17.18 ± 0.51 ^b^	16.15 ± 0.60 ^bc^	12.11 ± 1.08 ^d^	14.85 ± 0.45 ^c^	14.90 ± 0.48 ^c^	14.99 ± 0.36 ^c^	15.92 ± 0.43 ^bc^	16.48 ± 1.10 ^bc^
	*ΔE*	-	9.45 ± 0.64 ^b^	9.25 ± 0.88 ^b^	16.72 ± 1.53 ^a^	11.47 ± 0.77 ^b^	10.99 ± 0.71 ^b^	11.07 ± 0.52 ^b^	10.03 ± 1.15 ^b^	9.14 ± 1.43 ^b^
	C*	24.43 ± 0.79 ^a^	17.20 ± 0.51 ^b^	16.17 ± 0.60 ^bc^	12.17 ± 1.08 ^d^	14.89 ± 0.46 ^c^	14.91 ± 0.48 ^c^	15.02 ± 0.37 ^c^	15.95 ± 0.44 ^bc^	16.51 ± 1.11 ^bc^
	*h*°	275.35 ± 0.75 ^a^	272.98 ± 0.09 ^c^	273.05 ± 0.45 ^c^	84.22 ± 0.73 ^d^	274.42 ± 0.67 ^ab^	272.52 ± 0.14 ^c^	273.78 ± 0.43 ^bc^	273.67 ± 0.75 ^bc^	273.41 ± 0.41 ^bc^

Data represent mean ± standard deviation. Different letters within a row denote statistically significant differences among the samples (*p* < 0.05). CC: Control muffin, SC: Soy milk muffin, HC: Hazelnut milk muffin, WC: Walnut milk muffin, QC: Quinoa milk muffin, FC: Flaxseed milk muffin, CNC: Coconut milk muffin, OC: Oat milk muffin, AC: Almond milk muffin; VI: Volume index, SI: Symmetry index, UI: Uniformity index.

**Table 5:** Fatty acid composition of muffin samples.

Fatty Acids (%)	CC	SC	HC	WC	QC	FC	CNC	OC	AC
C14:0	0.166 ± 0.004 ^b^	0.080 ± 0.003 ^d^	0.075 ± 0.002 ^d^	0.077 ± 0.002 ^d^	0.078 ± 0.005 ^d^	0.793 ± 0.010 ^a^	0.126 ± 0.003 ^c^	0.076 ± 0.006 ^d^	0.084 ± 0.002 ^d^
C16:0	8.076 ± 0.022 ^a^	6.623 ± 0.011 ^bc^	6.583 ± 0.012 ^d^	6.600 ± 0.007 ^cd^	6.619 ± 0.007 ^bcd^	6.603 ± 0.009 ^bcd^	6.595 ± 0.013 ^cd^	6.641 ± 0.012 ^b^	6.625 ± 0.023 ^bc^
C16:1	0.241 ± 0.011 ^a^	0.103 ± 0.013 ^cd^	0.130 ± 0.009 ^bcd^	0.134 ± 0.012 ^bc^	0.135 ± 0.007 ^b^	0.100 ± 0.014 ^d^	0.138 ± 0.008 ^b^	0.113 ± 0.009 ^bcd^	0.107 ± 0.013 ^bcd^
C18:0	4.055 ± 0.141 ^a^	3.576 ± 0.018 ^b^	3.578 ± 0.013 ^b^	3.572 ± 0.062 ^b^	3.584 ± 0.017 ^b^	3.599 ± 0.012 ^b^	3.573 ± 0.018 ^b^	3.581 ± 0.014 ^b^	3.563 ± 0.009 ^b^
C18:1	35.303 ± 0.199 ^a^	35.051 ± 0.201 ^a^	35.081 ± 0.730 ^a^	34.643 ± 0.039 ^a^	34.992 ± 0.129 ^a^	34.910 ± 0.334 ^a^	35.097 ± 0.210 ^a^	34.779 ± 0.548 ^a^	35.288 ± 0.315 ^a^
C18:2	50.331 ± 0.205 ^c^	53.030 ± 0.133 ^ab^	52.745 ± 0.103 ^b^	53.210 ± 0.220 ^a^	53.071 ± 0.037 ^ab^	52.853 ± 0.112 ^ab^	52.734 ± 0.177 ^b^	53.039 ± 0.052 ^ab^	52.773 ± 0.187 ^b^
C18:3	0.134 ± 0.011 ^b^	0.112 ± 0.026 ^b^	0.081 ± 0.022 ^b^	0.341 ± 0.054 ^a^	0.082 ± 0.018 ^b^	0.309 ± 0.011 ^a^	0.081 ± 0.017 ^b^	0.074 ± 0.003 ^b^	0.076 ± 0.025 ^b^
C20:0	0.236 ± 0.021 ^a^	0.248 ± 0.014 ^a^	0.245 ± 0.009 ^a^	0.256 ± 0.019 ^a^	0.229 ± 0.025 ^a^	0.250 ± 0.017 ^a^	0.230 ± 0.019 ^a^	0.248 ± 0.014 ^a^	0.255 ± 0.007 ^a^
C22:0	0.642 ± 0.009 ^b^	0.693 ± 0.016 ^a^	0.688 ± 0.013 ^a^	0.679 ± 0.019 ^ab^	0.683 ± 0.009 ^ab^	0.675 ± 0.023 ^ab^	0.710 ± 0.011 ^a^	0.677 ± 0.015 ^ab^	0.684 ± 0.012 ^a^
C22:1	0.107 ± 0.014 ^a^	<LOD	<LOD	<LOD	<LOD	<LOD	<LOD	<LOD	<LOD
C24:0	0.239 ± 0.010 ^d^	0.265 ± 0.006 ^abc^	0.258 ± 0.006 ^bcd^	0.249 ± 0.012 ^cd^	0.284 ± 0.008 ^a^	0.275 ± 0.006 ^ab^	0.272 ± 0.012 ^abc^	0.253 ± 0.008 ^bcd^	0.257 ± 0.004 ^bcd^
SFA	13.399 ± 0.111 ^a^	11.494 ± 0.015 ^bc^	11.438 ± 0.025 ^c^	11.451 ± 0.049 ^c^	11.707 ± 0.340 ^bc^	11.977 ± 0.346 ^b^	11.569 ± 0.137 ^bc^	11.491 ± 0.013 ^bc^	11.492 ± 0.071 ^bc^
MUFA	35.636 ± 0.202 ^a^	35.250 ± 0.116 ^abcd^	35.648 ± 0.105 ^a^	34.805 ± 0.093 ^d^	35.133 ± 0.145 ^bcd^	34.969 ± 0.268 ^cd^	35.352 ± 0.094 ^abc^	35.249 ± 0.082 ^abcd^	35.516 ± 0.204 ^ab^
PUFA	50.462 ± 0.212 ^d^	53.222 ± 0.076 ^ab^	52.806 ± 0.053 ^c^	53.500 ± 0.093 ^a^	53.165 ± 0.040 ^b^	53.227 ± 0.070 ^ab^	52.791 ± 0.127 ^c^	53.138 ± 0.044 ^b^	52.814 ± 0.165 ^c^
TUFA	86.098 ± 0.414 ^b^	88.471 ± 0.191 ^a^	88.455 ± 0.097 ^a^	88.305 ± 0.184 ^a^	88.298 ± 0.181 ^a^	88.196 ± 0.231 ^a^	88.143 ± 0.113 ^a^	88.387 ± 0.114 ^a^	88.329 ± 0.122 ^a^

Data represent mean ± standard deviation. Different letters within a row denote statistically significant differences among the samples (*p* < 0.05). CC: Control muffin, SC: Soy milk muffin, HC: Hazelnut milk muffin, WC: Walnut milk muffin, QC: Quinoa milk muffin, FC: Flaxseed milk muffin, CNC: Coconut milk muffin, OC: Oat milk muffin, AC: Almond milk muffin; SFA: Saturated Fatty Acid, MUFA: Monounsaturated Fatty Acid, PUFA: Polyunsaturated Fatty Acid, TUFA: Total Unsaturated Fatty Acid.

**Table 6:** Total phenolic content and antioxidant activities of milk samples.

	Undigested	Simulated Gastric Digestion	Simulated Intestinal Digestion
TPC (mg GAE/g)			
CM	7.876 ± 0.704 ^a,A^	2.377 ± 0.208 ^c,B^	2.005 ± 0.020 ^b,B^
SM	1.755 ± 0.002 ^c,B^	3.232 ± 0.231 ^bc,A^	1.951 ± 0.032 ^b,B^
HM	0.181 ± 0.016 ^e,C^	0.629 ± 0.045 ^d,B^	1.117 ± 0.021 ^ef,A^
WM	4.623 ± 0.009 ^b,A^	4.933 ± 0.218 ^a,A^	3.579 ± 0.021 ^a,B^
QM	0.683 ± 0.059 ^de,B^	2.803 ± 0.392 ^c,A^	1.576 ± 0.020 ^c,B^
FM	0.850 ± 0.198 ^cde,B^	4.024 ± 0.611 ^ab,A^	1.217 ± 0.074 ^e,B^
CNM	0.065 ± 0.023 ^e,B^	0.413 ± 0.153 ^d,B^	1.081 ± 0.045 ^f,A^
OM	1.530 ± 0.123 ^cd,A^	1.010 ± 0.045 ^d,B^	1.406 ± 0.004 ^d,A^
AM	0.303 ± 0.019 ^e,B^	0.764 ± 0.045 ^d,A^	0.332 ± 0.017 ^g,B^
CUPRAC (µmol TE/g)			
CM	1.454 ± 0.102 ^a,A^	0.534 ± 0.019 ^b,B^	0.665 ± 0.005 ^b,B^
SM	0.040 ± 0.014 ^c,C^	0.308 ± 0.007 ^d,B^	0.550 ± 0.005 ^d,A^
HM	0.065 ± 0.068 ^c,B^	0.150 ± 0.015 ^f,B^	0.432 ± 0.019 ^f,A^
WM	1.489 ± 0.001 ^a,B^	1.220 ± 0.012 ^a,C^	1.693 ± 0.009 ^a,A^
QM	0.077 ± 0.022 ^c,C^	0.301 ± 0.006 ^d,B^	0.605 ± 0.003 ^c,A^
FM	0.229 ± 0.095 ^bc,B^	0.314 ± 0.009 ^d,AB^	0.488 ± 0.006 ^e,A^
CNM	0.038 ± 0.001 ^c,C^	0.442 ± 0.018 ^c,B^	0.646 ± 0.003 ^b,A^
OM	0.393 ± 0.103 ^b,A^	0.226 ± 0.006 ^e,A^	0.406 ± 0.013 ^f,A^
AM	0.041 ± 0.001 ^c,B^	0.341 ± 0.003 ^d,A^	0.287 ± 0.007 ^g,B^
DPPH (µmol TE/g)			
CM	175.450 ± 31.173 ^a,A^	24.618 ± 4.784 ^bc,B^	0.349 ± 0.007 ^e,B^
SM	28.075 ± 1.304 ^cd,A^	15.604 ± 1.533 ^c,B^	2.257 ± 0.011 ^a,C^
HM	54.392 ± 0.747 ^bc,A^	16.867 ± 2.120 ^c,B^	2.253 ± 0.019 ^a,C^
WM	90.426 ± 0.570 ^b,B^	152.967 ± 2.731 ^a,A^	0.621 ± 0.024 ^d,C^
QM	74.594 ± 0.288 ^b,C^	20.445 ± 2.017 ^c,B^	2.195 ± 0.008 ^b,A^
FM	84.034 ± 2.125 ^b,A^	35.190 ± 4.132 ^b,B^	1.846 ± 0.002 ^c,C^
CNM	50.689 ± 0.876 ^bc,A^	15.205 ± 1.014 ^c,B^	2.232 ± 0.010 ^ab,C^
OM	7.963 ± 1.207 ^d,B^	20.339 ± 0.761 ^c,A^	2.212 ± 0.016 ^ab,C^
AM	16.534 ± 0.502 ^cd,A^	18.905 ± 3.849 ^c,A^	1.847 ± 0.006 ^c,B^

Different lowercase letters within a column denote statistically significant differences (*p* < 0.05) among TPC, CUPRAC, and DPPH values of the milk samples, as determined by the Tukey test. Uppercase letters in each row indicate significant variations (*p* < 0.05) in total phenolic content across the simulated digestion phases. CM: Cow milk, SM: Soy milk, HM: Hazelnut milk, WM: Walnut milk, QM: Quinoa milk, FM: Flaxseed milk, CNM: Coconut milk, OM: Oat milk, AM: Almond milk.

**Table 7:** Total phenolic content and antioxidant activities of muffin samples.

	Undigested	Simulated Gastric Digestion	Simulated Intestinal Digestion
TPC (mg GAE/g)			
CC	2.080 ± 0.090 ^bcd,B^	2.066 ± 0.023 ^a,B^	3.499 ± 0.024 ^b,A^
SC	1.619 ± 0.166 ^cde,A^	1.428 ± 0.037 ^b,A^	0.336 ± 0.013 ^h,B^
HC	2.118 ± 0.388 ^bcd,A^	1.374 ± 0.028 ^b,AB^	0.719 ± 0.016 ^g,B^
WC	0.978 ± 0.032 ^e,B^	1.177 ± 0.174 ^b,AB^	1.578 ± 0.012 ^f,A^
QC	2.875 ± 0.040 ^ab,A^	1.414 ± 0.046 ^b,C^	2.491 ± 0.005 ^d,B^
FC	1.227 ± 0.210 ^de,B^	1.246 ± 0.044 ^b,B^	3.677 ± 0.016 ^a,A^
CNC	3.589 ± 0.074 ^a,A^	1.297 ± 0.059 ^b,C^	2.360 ± 0.021 ^e,B^
OC	2.483 ± 0.490 ^bc,A^	1.435 ± 0.026 ^b,AB^	0.337 ± 0.014 ^h,B^
AC	2.118 ± 0.076 ^bcd,B^	1.376 ± 0.007 ^b,C^	2.610 ± 0.011 ^c,A^
CUPRAC (µmol TE/g)			
CC	0.020 ± 0.008 ^cd,C^	0.884 ± 0.007 ^a,A^	0.633 ± 0.015 ^b,B^
SC	0.080 ± 0.017 ^ac,C^	0.439 ± 0.022 ^d,B^	0.516 ± 0.011 ^cd,A^
HC	0.116 ± 0.025 ^a,B^	0.492 ± 0.010 ^c,A^	0.444 ± 0.013 ^e,A^
WC	0.090 ± 0.022 ^ab,C^	0.389 ± 0.013 ^e,B^	0.472 ± 0.021 ^de,A^
QC	0.105 ± 0.014 ^ab,C^	0.394 ± 0.007 ^e,B^	0.479 ± 0.012 ^cde,A^
FC	0.091 ± 0.020 ^ab,B^	0.476 ± 0.006 ^cd,A^	0.531 ± 0.015 ^c,A^
CNC	0.047 ± 0.007 ^bcd,C^	0.380 ± 0.003 ^e,B^	0.467 ± 0.010 ^de,A^
OC	0.093 ± 0.012 ^ab,C^	0.496 ± 0.008 ^c,A^	0.371 ± 0.014 ^f,B^
AC	0.002 ± 0.000 ^d,C^	0.715 ± 0.006 ^b,B^	0.888 ± 0.019 ^a,A^
DPPH (µmol TE/g)			
CC	48.223 ± 3.217 ^b,A^	35.048 ± 1.191 ^a,B^	1.584 ± 0.005 ^f,C^
SC	43.515 ± 3.955 ^bc,A^	10.625 ± 0.037 ^c,B^	1.853 ± 0.005 ^a,C^
HC	60.528 ± 0.290 ^a,A^	14.510 ± 0.893 ^bc,B^	1.749 ± 0.007 ^c,C^
WC	62.914 ± 1.753 ^a,A^	18.605 ± 4.130 ^b,B^	1.730 ± 0.010 ^cd,C^
QC	20.942 ± 5.478 ^e,A^	15.159 ± 1.439 ^bc,B^	1.715 ± 0.004 ^d,C^
FC	62.425 ± 0.415 ^a,A^	10.361 ± 1.153 ^c,B^	1.721 ± 0.008 ^d,C^
CNC	40.998 ± 0.013 ^bcd,A^	15.430 ± 3.039 ^bc,B^	1.681 ± 0.003 ^e,C^
OC	31.709 ± 0.982 ^de,A^	0.390 ± 0.124 ^d,B^	1.826 ± 0.007 ^b,B^
AC	34.315 ± 3.414 ^cd,A^	18.763 ± 1.798 ^b,B^	1.752 ± 0.007 ^c,C^

Lowercase letters within each column indicate statistically significant differences (*p* < 0.05) among TPC, CUPRAC, and DPPH values of the muffin samples, as determined by Tukey’s test. Uppercase letters in each row denote significant variations (*p* < 0.05) in total phenolic content across the simulated digestion stages. CC: Control muffin, SC: Soy milk muffin, HC: Hazelnut milk muffin, WC: Walnut milk muffin, QC: Quinoa milk muffin, FC: Flaxseed milk muffin, CNC: Coconut milk muffin, OC: Oat milk muffin, AC: Almond milk muffin.

**Table 8:** Amino acid profile of muffin samples (g/100 g).

Amino Acids	CC	SC	HC	WC	QC	FC	CNC	OC	AC
Acidic amino acids									
Aspartic Acid	0.065 ± 0.002 ^c^	0.007 ± 0.000 ^g^	0.005 ± 0.000 ^g^	0.120 ± 0.002 ^b^	0.224 ± 0.003 ^a^	0.056 ± 0.002 ^d^	0.017 ± 0.000 ^f^	0.037 ± 0.001 ^e^	0.067 ± 0.004 ^c^
Glutamic Acid	0.476 ± 0.019 ^d^	0.061 ± 0.003 ^e^	0.032 ± 0.004 ^e^	1.895 ± 0.035 ^a^	1.850 ± 0.126 ^a^	1.238 ± 0.092 ^b^	0.147 ± 0.003 ^e^	0.353 ± 0.028 ^d^	0.873 ± 0.019 ^c^
Basic amino acids									
Arginine	1.007 ± 0.055 ^d^	0.203 ± 0.007 ^fg^	0.131 ± 0.005 ^g^	1.928 ± 0.047 ^a^	1.780 ± 0.111 ^b^	1.398 ± 0.052 ^c^	0.339 ± 0.011 ^f^	0.614 ± 0.009 ^e^	1.035 ± 0.018 ^d^
Histidine	0.321 ± 0.004 ^a^	0.098 ± 0.006 ^f^	0.002 ± 0.000 ^g^	0.313 ± 0.012 ^ab^	0.318 ± 0.018 ^a^	0.291 ± 0.009 ^bc^	0.130 ± 0.008 ^e^	0.211 ± 0.007 ^d^	0.277 ± 0.003 ^c^
Lysine	0.114 ± 0.005 ^b^	0.010 ± 0.000 ^e^	0.006 ± 0.000 ^e^	0.246 ± 0.004 ^a^	0.228 ± 0.018 ^a^	0.128 ± 0.005 ^b^	0.022 ± 0.000 ^e^	0.045 ± 0.001 ^d^	0.094 ± 0.002 ^c^
Neutral amino acids									
Alanine	0.136 ± 0.003 ^bc^	0.022 ± 0.002 ^e^	0.011 ± 0.000 ^e^	0.265 ± 0.006 ^a^	0.262 ± 0.019 ^a^	0.165 ± 0.023 ^b^	0.032 ± 0.001 ^e^	0.073 ± 0.002 ^d^	0.131 ± 0.011 ^c^
Serine	<LOD	<LOD	0.003 ± 0.000 ^a^	<LOD	<LOD	<LOD	<LOD	<LOD	0.004 ± 0.000 ^a^
Asparagine	0.072 ± 0.006 ^b^	0.010 ± 0.000 ^de^	0.007 ± 0.000 ^e^	0.099 ± 0.006 ^a^	0.096 ± 0.002 ^a^	0.081 ± 0.007 ^b^	0.019 ± 0.000 ^d^	0.039 ± 0.001 ^c^	0.071 ± 0.003 ^b^
Glutamine	0.007 ± 0.000 ^d^	<LOD	0.026 ± 0.002 ^a^	0.024 ± 0.001 ^a^	0.026 ± 0.001 ^a^	0.017 ± 0.001 ^b^	0.002 ± 0.000 ^e^	0.004 ± 0.000 ^e^	0.011 ± 0.001 ^c^
Glycine	0.039 ± 0.003 ^d^	<LOD	0.006 ± 0.000 ^f^	0.161 ± 0.008 ^a^	0.164 ± 0.006 ^a^	0.087 ± 0.005 ^b^	0.006 ± 0.000 ^f^	0.017 ± 0.001 ^e^	0.054 ± 0.001 ^c^
Threonine	0.050 ± 0.004 ^d^	<LOD	<LOD	0.153 ± 0.009 ^a^	0.150 ± 0.009 ^a^	0.099 ± 0.005 ^b^	0.015 ± 0.000 ^f^	0.034 ± 0.001 ^e^	0.069 ± 0.003 ^c^
Tyrosine	0.048 ± 0.003 ^c^	0.006 ± 0.000 ^e^	0.003 ± 0.000 ^e^	0.120 ± 0.004 ^a^	0.118 ± 0.003 ^a^	0.079 ± 0.005 ^b^	0.016 ± 0.001 ^d^	0.022 ± 0.001 ^d^	0.048 ± 0.004 ^c^
Cystine	0.061 ± 0.001 ^a^	0.026 ± 0.001 ^de^	0.020 ± 0.002 ^f^	0.033 ± 0.001 ^b^	0.028 ± 0.000 ^cd^	0.024 ± 0.002 ^e^	0.025 ± 0.001 ^de^	0.031 ± 0.001 ^bc^	0.017 ± 0.000 ^f^
Valine	0.124 ± 0.005 ^c^	0.014 ± 0.001 ^ef^	0.007 ± 0.000 ^f^	0.392 ± 0.014 ^a^	0.378 ± 0.011 ^a^	0.215 ± 0.005 ^b^	0.031 ± 0.002 ^e^	0.059 ± 0.001 ^d^	0.138 ± 0.004 ^c^
Methionine	0.088 ± 0.002 ^b^	0.015 ± 0.000 ^f^	0.013 ± 0.000 ^f^	0.102 ± 0.007 ^a^	0.089 ± 0.005 ^b^	0.068 ± 0.003 ^c^	0.020 ± 0.001 ^f^	0.034 ± 0.002 ^e^	0.053 ± 0.002 ^d^
Norvaline	0.004 ± 0.000 ^b^	0.003 ± 0.000 ^b^	<LOD	<LOD	<LOD	<LOD	<LOD	0.006 ± 0.000 ^a^	<LOD
Trptophan	0.007 ± 0.000 ^c^	<LOD	0.003 ± 0.000 ^d^	0.021 ± 0.002 ^a^	0.017 ± 0.001 ^b^	0.021 ± 0.001 ^a^	0.004 ± 0.000 ^d^	0.003 ± 0.000 ^d^	0.019 ± 0.001 ^b^
Phenylalanine	0.105 ± 0.012 ^c^	0.016 ± 0.000 ^e^	0.011 ± 0.001 ^e^	0.288 ± 0.016 ^a^	0.278 ± 0.013 ^a^	0.175 ± 0.006 ^b^	0.028 ± 0.001 ^e^	0.059 ± 0.004 ^d^	0.124 ± 0.002 ^c^
Isoleucine	0.087 ± 0.005 ^d^	0.011 ± 0.001 ^f^	0.008 ± 0.001 ^f^	0.276 ± 0.005 ^a^	0.268 ± 0.010 ^a^	0.151 ± 0.006 ^b^	0.021 ± 0.000 ^f^	0.048 ± 0.001 ^e^	0.107 ± 0.006 ^c^
Leucine	0.330 ± 0.016 ^c^	0.051 ± 0.001 ^ef^	0.031 ± 0.001 ^f^	0.602 ± 0.014 ^a^	0.589 ± 0.053 ^a^	0.427 ± 0.011 ^b^	0.097 ± 0.003 ^e^	0.186 ± 0.010 ^d^	0.339 ± 0.001 ^c^
Hdroxyproline	<LOD	0.047 ± 0.001 ^e^	0.031 ± 0.004 ^f^	0.225 ± 0.001 ^a^	0.200 ± 0.010 ^b^	0.050 ± 0.002 ^e^	0.067 ± 0.003 ^d^	0.078 ± 0.003 ^c^	0.065 ± 0.002 ^d^
Sarcosine	0.052 ± 0.002 ^bc^	0.039 ± 0.001 ^d^	0.041 ± 0.002 ^d^	0.104 ± 0.008 ^a^	0.101 ± 0.001 ^a^	<LOD	0.044 ± 0.002 ^cd^	0.041 ± 0.001 ^d^	0.052 ± 0.001 ^b^
Proline	0.037 ± 0.000 ^d^	0.087 ± 0.008 ^a^	0.087 ± 0.002 ^a^	0.032 ± 0.000 ^de^	0.023 ± 0.001 ^ef^	0.017 ± 0.000 ^f^	0.060 ± 0.001 ^c^	0.093 ± 0.001 ^a^	0.071 ± 0.005 ^b^

Data represent mean ± standard deviation. Different letters within a row denote statistically significant differences among the samples (*p* < 0.05). “<LOD”: below the limit of detection. CC: Control muffin, SC: Soy milk muffin, HC: Hazelnut milk muffin, WC: Walnut milk muffin, QC: Quinoa milk muffin, FC: Flaxseed milk muffin, CNC: Coconut milk muffin, OC: Oat milk muffin, AC: Almond milk muffin.

## Data Availability

The original contributions presented in this study are included in the article. Further inquiries can be directed to the corresponding author.

## Supplementary Materials

The following supporting information can be downloaded at: https://www.mdpi.com/article/10.3390/foods14233989/s1, Supplementary Table S1: Changes in physicochemical properties of muffin samples throughout shelf life. Supplementary Table S2: Microbiological analysis results of muffin samples throughout shelf life. Supplementary Table S3: Explained variance of the first seven principal components.
