# Supplementary material for: Evaluation of Plant-Based Milks in Vegan Muffins: Functional, Structural, Rheological and Nutritional Characterization
Source: Foods. 2025 Nov 21;14(23):3989. doi: 10.3390/foods14233989 (PMC12692624; doi:10.3390/foods14233989)
Supplement: Supplementary file 1 [file foods-14-03989-s001.zip › foods-3976611-supplementary.pdf]

Supplementary Materials:

Supplementary Table S1. Changes in physicochemical properties of muffin samples throughout shelf life

			CC	SC	HC	WC	QC	FC	CNC	OC	AC
**Chemical properties**											
Moisture (%)		Day 0	14.45±0,00^{c}	14.91±0.08^{b}	14.45±0.03^{c}	14.86±0.07^{b}	16.18±0.06^{a}	14.62±0.02^{c}	14.82±0.02^{b}	14.20±0.00^{d}	13.72±0.05^{e}
		Day 15	17.02±0.43^{c}	18.42±0.39^{bc}	19.94±0.35^{a}	18.97±0.34^{ab}	19.87±0.51^{ab}	19.34±0.35^{ab}	19.51±0.46^{ab}	17.38±0.25^{c}	18.28±0.45^{bc}
		Day 30	10.07±0.02^{e}	9.17±0.02^{f}	12.35±0.03^{c}	12.53±0.03^{b}	12.55±0.03^{b}	15.71±0.04^{a}	12.59±0.01^{b}	12.23±0.04^{d}	12.18±0.01^{d}
Ash (%)		Day 0	0.73±0.03^{bc}	0.82±0.02^{b}	0.73±0.03^{bc}	0.72±0.02^{c}	0.97±0.03^{a}	0.82±0.02^{a}	0.80±0.01^{bc}	0.93±0.02^{a}	0.76±0.01^{bc}
		Day 15	0.92±0.02^{a}	0.84±0.19^{ab}	0.82±0.03^{ab}	0.90±0.05^{ab}	0.73±0.14^{bc}	0.88±0.21^{ab}	0.81±0.12^{ab}	0.63±0.06^{c}	0.85±0.22^{ab}
		Day 30	0.83±0.02^{bc}	0.57±0.06^{d}	0.94±0.03^{b}	0.74±0.04^{c}	0.86±0.02^{bc}	0.79±0.03^{c}	0.75±0.02^{c}	1.18±0.10^{a}	1.11±0.03^{a}
Fat (%)		Day 0	22.21±0.02^{a}	20.36±0.04^{c}	20.36±0.05^{c}	20.82±0.06^{b}	19.61±0.01^{e}	20.23±0.03^{c}	20.06±0.02^{d}	20.76±0.02^{b}	19.92±0.04^{d}
		Day 15	20.27±0.45^{a}	20.20±0.33^{a}	19.28±0.55^{ab}	19.92±0.47^{ab}	18.63±0.32^{bc}	19.87±0.24^{ab}	18.44±0.45^{bc}	16.53±0.27^{c}	18.88±0.41^{bc}
		Day 30	16.66±0.07^{g}	21.73±0.02^{b}	21.67±0.36^{b}	21.84±0.02^{a}	20.94±0.04^{e}	20.94±0.04^{e}	21.21±0.01^{c}	17.99±0.04^{f}	21.05±0.04^{e}
**Texture**											
Hardness (gf)		Day 0	1284.74±104.18^{bc}	1616.01±205.37^{a}	1584.69±141.61^{a}	976.59±68.83^{d}	1158.40±82.19^{c}	1015.00±3.33^{cd}	1416.21±143.37^{ab}	1459.38±101.54^{ab}	1404.69±103.67^{ab}
		Day 15	2696.99±92.60^{c}	nm	3641.62±335.69^{b}	nm	nm	nm	4153.08±189.56^{a}	nm	4116.30±127.89^{a}
		Day 30	nm	nm	nm	nm	nm	nm	nm	nm	nm
Springiness		Day 0	0.91±0.01^{a}	0.88±0.01^{b}	0.88±0.02^{b}	0.86±0.00^{c}	0.88±0.01^{b}	0.84±0.01^{d}	0.89±0.01^{ab}	0.89±0.01^{ab}	0.89±0.03^{ab}
		Day 15	0.82±0.01^{a}	nm	0.73±0.03^{c}	nm	nm	nm	0.75±0.00^{bc}	nm	0.76±0.02^{bc}
		Day 30	nm	nm	nm	nm	nm	nm	nm	nm	nm
Cohesiveness		Day 0	0.71±0.00^{a}	0.58±0.00^{c}	0.59±0.03^{c}	0.64±0.02^{b}	0.62±0.01^{bc}	0.60±0.01^{c}	0.60±0.03^{c}	0.62±0.01^{bc}	0.60±0.01^{c}
		Day 15	0.49±0.04^{a}	nm	0.35±0.01^{c}	nm	nm	nm	0.33±0.01^{c}	nm	0.40±0.01^{b}
		Day 30	nm	nm	nm	nm	nm	nm	nm	nm	nm
Chewiness		Day 0	828.54±70.46^{a}	823.56±117.80^{a}	832.87±116.75^{a}	534.95±23.83^{d}	633.71±33.72^{cd}	513.33±9.88^{d}	755.78±74.46^{bc}	808.05±64.91^{ab}	748.21±72.95^{bc}
		Day 15	1082.17±47.76^{b}	nm	926.20±129.77^{b}	nm	nm	nm	1028.37±15.62^{b}	nm	1236.76±49.37^{a}
		Day 30	nm	nm	nm	nm	nm	nm	nm	nm	nm
Resilience		Day 0	0.33±0.00^{a}	0.25±0.00^{c}	0.26±0.02^{c}	0.30±0.02^{ab}	0.29±0.01^{b}	0.27±0.01^{bc}	0.27±0.02^{bc}	0.28±0.01^{bc}	0.26±0.01^{c}
		Day 15	0.18±0.02^{a}	nm	0.14±0.00^{c}	nm	nm	nm	0.13±0.00^{c}	nm	0.16±0.00^{b}
		Day 30	nm	nm	nm	nm	0.17±0.01^{a}	nm	nm	nm	nm
**Color**											
Crust	*L**	Day 0	58.98±3.05^{c}	64.65±2.77^{ab}	62.89±2.19^{bc}	65.12±1.17^{ab}	65.27±2.35^{ab}	64.40±2.17^{ab}	67.46±2.86^{ab}	63.67±1.06^{bc}	69.12±1.96^{a}
		Day 15	54.13±3.09^{e}	65.27±4.94^{ab}	58.63±1.66^{cd}	62.92±2.20^{ab}	66.40±4.62^{a}	66.30±4.17^{a}	62.37±1.11^{abc}	61.35±3.66^{bc}	64.23±2.99^{ab}
		Day 30	56.11±4.62^{b}	64.04±4.89^{a}	61.41±0.88^{ab}	64.15±3.90^{a}	67.61±2.94^{a}	65.33±2.20^{a}	64.23±2.54^{a}	61.46±7.44^{ab}	66.14±3.75^{a}
	*a**	Day 0	12.48±1.88^{a}	6.04±3.42^{b}	6.44±2.67^{ab}	4.74±0.79^{b}	3.91±2.97^{b}	4.45±3.65^{b}	4.95±2.91^{b}	5.03±2.45^{b}	3.21±1.83^{b}
		Day 15	14.10±1.08^{a}	5.91±2.79^{cde}	7.94±2.93^{c}	5.25±1.44^{cde}	4.49±2.73^{de}	5.04±2.40^{de}	5.79±2.44^{cde}	5.59±2.09^{cde}	6.72±1.96^{cd}
		Day 30	12.24±0.62^{a}	6.47±2.47^{b}	4.52±2.74^{b}	5.32±2.11^{b}	4.12±1.78^{b}	5.22±1.52^{b}	3.93±1.96^{b}	5.64±2.35^{b}	4.55±2.11^{b}
	*b**	Day 0	30.99±2.86^{a}	28.96±0.47^{ab}	28.75±0.90^{ab}	24.57±0.92^{b}	24.49±2.61^{b}	26.34±2.64^{b}	27.82±1.85^{ab}	27.74±1.13^{ab}	25.92±2.26^{b}
		Day 15	30.32±2.89^{a}	28.56±2.41^{ab}	27.41±1.71^{ab}	23.04±2.68de	26.47±2.40^{bc}	26.47±1.91^{bc}	26.68±1.47^{bc}	26.89±1.26^{bc}	29.49±1.55^{ab}
		Day 30	33.28±2.65^{a}	29.16±1.00^{b}	28.56±2.01^{b}	23.60±2.95^{c}	27.68±1.61^{b}	27.40±1.57^{b}	27.08±1.37^{bc}	27.54±1.19^{b}	28.21±2.03^{b}
Crumb	*L**	Day 0	75.03±0.85^{a}	69.58±0.55^{ab}	72.02±1.13^{b}	64.69±1.16^{c}	69.38±0.82^{b}	70.46±0.84^{ab}	69.98±0.63^{ab}	70.62±1.82^{ab}	71.43±1.24^{ab}
		Day 15	75.97±1.74^{a}	71.30±0.71^{bcd}	70.54±0.57^{cde}	65.80±1.18^{f}	72.71±0.78^{b}	71.60±1.59^{bc}	69.81±0.55^{de}	69.10±0.81^{e}	71.31±1.14^{bcd}
		Day 30	73.75±2.50^{a}	71.79±1.29^{a}	68.23±1.16^{ab}	66.97±0.75^{ab}	73.42±0.62^{a}	72.49±0.76^{a}	67.84±2.64^{ab}	62.33±3.16^{b}	67.91±1.70^{ab}
	*a**	Day 0	-2.28±0.29^{d}	-0.90±0.04^{bc}	-0.86±0.15^{bc}	1.22±0.10^{a}	-1.15±0.19^{c}	-0.66±0.05^{b}	-0.99±0.13^{bc}	-1.02±0.22^{bc}	-0.99±0.16^{bc}
		Day 15	-0.99±0.43^{de}	-1.28±0.29^{ef}	-1.03±0.25^{de}	2.86±0.24^{a}	-1.58±0.21^{f}	-0.32±0.49^{c}	-1.31±0.07^{ef}	-1.29±0.38^{ef}	-0.90±0.22^{de}
		Day 30	-0.76±0.78^{b}	-2.55±3.95^{b}	-0.76±0.33^{b}	2.78±0.19^{a}	-1.16±0.11^{b}	-0.01±0.22^{b}	-1.05±0.19^{b}	-1.19±0.06^{b}	-0.43±0.75^{b}
	*b**	Day 0	24.33±0.80^{a}	17.18±0.51^{b}	16.15±0.60^{bc}	12.11±1.08^{d}	14.85±0.45^{c}	14.90±0.48^{c}	14.99±0.36^{c}	15.92±0.43^{bc}	16.48±1.10^{bc}

Day 15	24.24±0.55^{a}	18.85±0.64^{cde}	18.41±0.84^{de}	14.32±0.45^{g}	18.24±0.44^{e}	18.34±0.63^{de}	19.05±0.42^{cd}	18.87±0.63^{cde}	19.78±0.49^{b}
Day 30	23.51±0.96^{a}	17.87±0.42^{c}	16.66±2.21^{c}	13.97±0.27^{d}	18.03±0.47^{bc}	17.29±0.39^{c}	18.06±0.52^{bc}	17.80±0.40^{c}	19.55±0.57^{b}

Results are displayed as the means ± standard deviation. Means followed by different letters in the same row are significantly different ($p < 0.05$). CC: Control muffin, SC: Soy milk muffin, HC: Hazelnut milk muffin, WC: Walnut milk muffin, QC: Quinoa milk muffin, FC: Flaxseed milk muffin, CNC: Coconut milk muffin, OC: Oat milk muffin, AC: Almond milk muffin

Supplementary Table S2. Microbiological analysis results of muffin samples throughout shelf life

	Aerobic Plate Count (APC), CFU/g			*E. coli*, CFU/g			Coliforms, CFU/g			*S. aureus*, CFU/g			Yeasts and Molds, CFU/g			*Salmonella* spp., CFU/g		
	Day 0	Day 15	Day 30	Day 0	Day 15	Day 30	Day 0	Day 15	Day 30	Day 0	Day 15	Day 30	Day 0	Day 15	Day 30	Day 0	Day 15	Day 30
**CC**	$2.7x10^{2}$ ^{d,B}	$7.5x10^{2}$ ^{d,A}	$2.6x10^{2}$ ^{f,B}	<10	<10	<10	<10	<10	<10	<10	<10	<10	<10	<10	<10	<LOD	<LOD	<LOD
**SC**	$2.0x10^{2}$ ^{e,C}	$9.2x10^{3}$ ^{a,A}	$1.2x10^{3}$ ^{b,B}	<10	<10	<10	<10	<10	<10	<10	<10	<10	<10	<10	<10	<LOD	<LOD	<LOD
**HC**	$3.4x10^{2}$ ^{c,C}	$7.8x10^{2}$ ^{d,A}	$5.5x10^{2}$ ^{d,B}	<10	<10	<10	<10	<10	<10	<10	<10	<10	<10	<10	<10	<LOD	<LOD	<LOD
**WC**	$9.1x10^{2}$ ^{a,A}	$1.5x10^{2}$ ^{e,C}	$4.1x10^{2}$ ^{e,B}	<10	<10	<10	<10	<10	<10	<10	<10	<10	<10	<10	<10	<LOD	<LOD	<LOD
**QC**	$3.2x10^{2}$ ^{c,C}	$9.6x10^{2}$ ^{d,A}	$6.0x10^{2}$ ^{d,B}	<10	<10	<10	<10	<10	<10	<10	<10	<10	<10	<10	<10	<LOD	<LOD	<LOD
**FC**	$4.4x10^{2}$ ^{b,C}	$7.3x10^{2}$ ^{d,A}	$6.3x10^{2}$ ^{d,B}	<10	<10	<10	<10	<10	<10	<10	<10	<10	<10	<10	<10	<LOD	<LOD	<LOD
**CNC**	$2.8x10^{2}$ ^{d,C}	$5.8x10^{2}$ ^{d,B}	$1.3x10^{3}$ ^{b,A}	<10	<10	<10	<10	<10	<10	<10	<10	<10	<10	<10	<10	<LOD	<LOD	<LOD
**OC**	$1.5x10^{2}$ ^{f,C}	$4.8x10^{3}$ ^{b,A}	$8.6x10^{2}$ ^{c,B}	<10	<10	<10	<10	<10	<10	<10	<10	<10	<10	<10	<10	<LOD	<LOD	<LOD
**AC**	$5.1x10^{2}$ ^{b,C}	$1.3x10^{3}$ ^{c,B}	$2.1x10^{3}$ ^{a,A}	<10	<10	<10	<10	<10	<10	<10	<10	<10	<10	<10	<10	<LOD	<LOD	<LOD

Results are displayed as the means ± standard deviation. Different lower case letters in the same column indicate the difference between samples. Different capital letters on the same row indicate the difference between days ($P<0.05$). CC: Control muffin, SC: Soy milk muffin, HC: Hazelnut milk muffin, WC: Walnut milk muffin, QC: Quinoa milk muffin, FC: Flaxseed milk muffin, CNC: Coconut milk muffin, OC: Oat milk muffin, AC: Almond milk muffin

Supplementary Table S3. Explained variance of the first seven principal components

Variables	PC1	PC2	PC3	PC4	PC5	PC6	PC7
Eigenvalue	31,503	25,339	7,640	5,676	5,212	4,598	3,353
Percentage %	32,477	26,123	7,876	5,852	5,373	4,740	3,456
Cumulative	32,477	58,600	66,476	72,328	77,701	82,441	85,898
K'	-0,662	-0,379	0,221	-0,515	-0,092	-0,181	-0,091
n'	0,503	-0,151	0,020	-0,220	-0,707	-0,159	-0,314
R2'	-0,184	0,293	-0,035	0,281	0,696	0,318	0,388
K''	-0,745	-0,135	0,291	-0,478	0,286	0,000	0,107
n''	0,470	0,495	-0,052	0,041	-0,123	0,347	-0,060
R2''	-0,560	-0,117	-0,007	0,078	-0,013	-0,061	-0,048
Tan(θ)	0,812	0,339	0,171	-0,031	-0,314	0,088	-0,087
Baking Loss	0,054	-0,061	-0,166	-0,265	-0,120	0,074	0,025
VI	0,905	-0,123	0,086	-0,040	0,131	-0,110	0,102
SI	0,090	-0,130	0,601	0,192	-0,163	-0,279	-0,042
UI	0,233	-0,011	0,296	0,178	0,349	0,239	-0,423
Hardness	-0,187	0,526	0,116	0,196	-0,047	0,722	0,069
Springiness	0,437	0,318	-0,087	0,187	0,240	0,408	0,225
Cohesiveness	0,845	0,150	-0,144	-0,013	0,044	-0,338	0,160
Chewiness	0,166	0,590	0,029	0,219	-0,002	0,641	0,131
Resilience	0,772	0,035	-0,146	-0,002	0,081	-0,444	0,187
Crust-L	-0,695	-0,137	0,231	0,246	0,127	-0,261	-0,092
Crust-a	0,786	0,049	-0,183	-0,131	0,094	0,187	-0,127
Crust-b	0,583	-0,434	-0,280	-0,098	0,025	0,135	-0,195
Crumb-L	0,721	-0,394	0,327	0,093	-0,050	0,108	-0,081
Crumb-a	-0,670	0,407	-0,516	0,195	0,023	-0,112	0,037
Crumb-b	0,929	-0,144	0,121	-0,038	0,068	0,087	0,050
Moisture	-0,324	0,410	0,278	-0,588	0,029	0,524	0,013
Ash	-0,356	-0,053	0,439	-0,553	-0,172	-0,155	0,165
Protein	0,941	0,266	-0,058	-0,085	0,107	0,010	-0,033
Total Carbohydrate	-0,828	-0,358	0,071	0,330	-0,074	-0,208	-0,044
TDF	0,058	-0,702	0,086	0,389	0,433	0,290	0,136
Fat	0,855	0,205	-0,431	-0,098	-0,065	-0,092	0,052
Energy	0,768	-0,016	-0,469	0,226	-0,060	-0,337	0,013
P	0,850	-0,062	0,033	-0,139	0,151	0,368	-0,039
Na	0,736	-0,178	0,055	-0,215	0,261	0,366	-0,186
Mg	-0,896	-0,180	-0,097	-0,007	-0,058	0,296	0,026
K	-0,606	-0,321	-0,135	0,208	-0,092	0,392	0,099
Ca	0,769	-0,573	-0,098	0,095	0,226	-0,079	0,008
TPC-Undigested-Muffin	0,023	-0,438	0,513	-0,539	0,145	-0,164	-0,165
TPC-Gastric-Muffin	0,913	0,001	0,137	-0,202	0,152	0,056	0,043
TPC-Intestinal-Muffin	0,373	0,478	0,555	0,281	-0,286	-0,032	-0,334
CUPRAC-Undigested-Muffin	-0,533	0,071	-0,233	-0,310	-0,250	0,430	0,299
CUPRAC-Gastric-Muffin	0,848	0,005	0,181	0,331	0,192	-0,220	0,034
CUPRAC-Intestinal-Muffin	0,280	0,041	0,376	0,664	0,362	-0,244	-0,106
DPPH-Undigested-Muffin	0,118	0,188	-0,610	0,506	-0,327	0,298	-0,121
DPPH-Gastric-Muffin	0,724	0,398	0,121	0,179	0,325	0,145	-0,096
DPPH-Intestinal-Muffin	-0,644	-0,433	-0,289	0,007	0,032	-0,046	0,327
Aspartic Acid	-0,236	0,763	0,463	-0,263	0,236	0,015	0,098
Glutamic Acid	-0,357	0,900	0,211	0,051	0,021	-0,068	0,040
Asparagine	-0,018	0,911	0,280	0,109	-0,012	-0,216	-0,022
Serine	-0,062	-0,346	0,187	0,689	0,509	-0,116	0,067
Glutamine	-0,338	0,558	0,115	0,214	0,184	0,310	0,117
Histidine	0,162	0,804	0,312	0,009	-0,094	-0,372	-0,029
Glycine	-0,343	0,912	0,191	-0,031	0,084	0,005	0,048
Threonine	-0,299	0,918	0,212	0,011	0,015	-0,107	0,022
Arginine	-0,169	0,944	0,208	0,051	-0,030	-0,148	0,032
Alanine	-0,168	0,943	0,216	-0,014	0,058	-0,116	0,030
Tyrosine	-0,248	0,943	0,205	-0,011	-0,003	-0,032	0,018

Cystine	0,831	0,394	-0,129	-0,347	-0,039	-0,018	0,028
Valine	-0,294	0,935	0,167	-0,037	0,061	-0,030	0,041
Methionine	0,166	0,961	0,147	0,007	0,028	-0,122	0,031
Norvaline	0,464	-0,264	-0,262	-0,452	-0,129	-0,303	0,277
Trptophan	-0,292	0,772	0,287	0,431	-0,059	-0,175	-0,079
Phenylalanine	-0,273	0,935	0,193	0,005	0,049	-0,062	0,037
Isoleucine	-0,301	0,930	0,176	-0,023	0,080	-0,039	0,040
Leucine	-0,152	0,939	0,243	0,042	0,012	-0,141	0,010
Lysine	-0,165	0,964	0,156	-0,039	0,090	-0,060	0,038
Hdroxyproline	-0,608	0,678	-0,031	-0,272	0,244	-0,089	0,066
Sarcosine	-0,193	0,594	-0,015	-0,359	0,652	-0,057	0,118
Proline	-0,052	-0,838	-0,320	-0,030	0,301	-0,147	0,185
C14:0	-0,004	0,194	0,178	0,387	-0,833	0,147	-0,169
C16:0	0,964	0,215	0,042	-0,107	0,040	0,014	0,001
C16:1	0,850	0,285	0,032	-0,259	0,168	0,034	-0,069
C18:0	0,926	0,224	0,044	-0,115	-0,002	0,070	-0,043
C18:1	0,361	-0,217	0,341	0,210	0,164	0,185	-0,068
C18:2	-0,970	-0,093	-0,099	0,005	-0,011	-0,036	0,073
C18:3	-0,128	0,686	-0,403	0,320	-0,431	0,074	-0,105
C20:0	-0,150	0,037	-0,338	0,399	-0,072	-0,232	0,311
C22:0	-0,599	-0,385	-0,023	-0,027	0,067	0,152	-0,203
C22:1	0,962	0,218	0,036	-0,100	0,038	0,044	-0,020
C24:0	-0,571	-0,068	0,507	-0,114	-0,274	0,275	-0,023
SFA	0,922	0,244	0,091	-0,007	-0,184	0,059	-0,075
MUFA	0,543	-0,554	0,231	0,100	0,316	-0,031	0,159
PUFA	-0,967	-0,026	-0,136	0,050	-0,111	-0,016	0,112
TUFA	-0,918	-0,260	-0,065	0,101	0,000	-0,031	0,198
Sensory-CrustColor	0,619	-0,278	0,019	0,244	-0,143	0,008	0,161
Sensory-InnerColor	0,617	-0,270	0,466	0,089	-0,107	0,160	0,290
Sensory-Flavor	0,563	-0,067	0,244	0,206	-0,364	-0,014	0,330
Sensory-Taste	0,432	0,064	0,193	0,086	-0,344	-0,091	0,561
Sensory-Appearance	0,520	-0,264	0,337	0,066	-0,334	0,097	0,475
Sensory-Softness	0,137	0,186	0,133	-0,077	-0,269	-0,044	0,771
Sensory-GeneralAcceptance	0,543	-0,053	0,274	0,090	-0,151	0,025	0,578
